# Mechanical properties of crystalline-amorphous composites: generalization of Hall–Petch and inverse Hall–Petch behaviors

**DOI:** 10.1093/nsr/nwaf336

**Published:** 2025-08-20

**Authors:** Zhibin Xu, Mengmeng Li, Yilong Han

**Affiliations:** Department of Physics, The Hong Kong University of Science and Technology, Hong Kong, China; Department of Physics, The Hong Kong University of Science and Technology, Hong Kong, China; Department of Physics, The Hong Kong University of Science and Technology, Hong Kong, China

**Keywords:** Hall–Petch and inverse Hall–Petch behaviors, crystalline-amorphous composite, ductile–brittle transition, elastic moduli, solid mechanics

## Abstract

The strength, $\sigma _{\rm y}$, of a polycrystal decreases with the mean grain diameter $D$ for $D\gtrsim 50$ atoms (i.e. Hall–Petch behavior) and increases for $D \lesssim 50$ (i.e. inverse Hall–Petch behavior). Our simulations generalize $\sigma _{\rm y}(D)$ to $\sigma _{\rm y}(D,l)$, where $l$ is the mean thickness of amorphous grain boundaries of crystalline-amorphous composites. The maximum strength is reached at $(D,l)\approx (50, 6)$ atoms for single-component face-centered-cubic solids and at $(D,l)\approx (50, 2)$ for bidispersed or body-centered-cubic solids because of the different activation stresses of dislocation motions. The results explain recent alloy experiments and provide a way to exceed the maximum strength of polycrystals. Ductility and elastic moduli are also measured in the broad $(D,l)$ space. In regimes without a strength-ductility trade-off, the maximum ductility and ductile–brittle transitions are identified. These results obtained in $(D,l)$ space are important in solid mechanics and can guide the fabrication of crystalline-amorphous composites with outstanding mechanical properties.

## INTRODUCTION

Polycrystalline materials, such as ceramics, metals and alloys, are ubiquitous in nature and industries. Their strength or yield stress, $\sigma _{\rm y}$, increases as the mean grain diameter $D$ decreases, i.e. the famous Hall–Petch (HP) behavior discovered in the 1950s [1,2]. This trend reverses at $D \lesssim 10$–$20\ {\rm nm}\approx 50$ atoms, i.e. the inverse Hall–Petch (IHP) behavior [3,4]. HP and IHP behaviors generally hold in all atomic and molecular polycrystals and have been intensively studied [5–8]. Aside from tuning $D$, a polycrystal’s strength can also be enhanced by increasing pressure [9] and decreasing temperature [8] or the initial dislocation density [8]. However, previous studies have focused on solid strengthening instead of generalizing HP and IHP behaviors by tuning structural parameters in a broad range. An important simulation study obtained a non-monotonic $\sigma _{\rm y}$ by adding twin boundaries [10]; this result is essentially covered by the HP and IHP behaviors because twin boundaries can be viewed as a special type of grain boundary (GB). Here we generalize the HP and IHP behaviors of $\sigma _{\rm y}(D)$ for polycrystals with mean GB thickness $l\approx 2$ atoms to $\sigma _{\rm y}(D,l)$ by tuning structural parameters $D$ and $l$.

Thick-GB ($l>2$) polycrystals are difficult to fabricate. Thus, the effect of $l$ on material properties remains unclear and poorly explored. In recent years, thick-GB polycrystals have been fabricated in glass-ceramics [11] and alloys that are often called crystalline-amorphous composites [12], dual-phase crystal-glass materials [13] or GB complexions [14]. Thick GBs are alternatively termed the ‘amorphous intergranular film phase’ (AIF phase) or ‘grain boundary complexion’ in the literature [15]. According to Xu *et al.* [15], ‘AIF (amorphous phase with a different composition between crystalline grains)’ $\subseteq$ ‘thick GB (amorphous phases between crystalline grains)’ $\subseteq$ ‘GB complexion (any phases between crystalline grains)’. Here, ‘$\subseteq$’ denotes the subset relationship. Such materials exhibit ultrahigh strength [11,16,17], superplasticity [18], ultralong fatigue life [19], hydrogen storage ability [12] and wear resistance [11,12]. They are often fabricated by complex trial-and-error processes, so the sizes of the crystalline and amorphous regions are difficult to control [13]. Thus, the effect of $l$ on mechanical properties has been poorly explored. Simulations of the effects of thick GBs on material properties are limited to Cu-based alloys within a narrow range of $l$ [20,21]. A recent simulation measured the strength of two-dimensional (2D) polycrystals with thick GBs; such solids only exhibited IHP behavior, and the maximum strength at the HP-IHP transition was not determined  [22]. Here, we systematically measure the strength of 3D solids so that both the HP and IHP behaviors can be generalized to the $(D,l)$ parameter space. To examine the effects of lattice symmetry and atomic composition, we compare four types of systems having different lattice structures and compositions. We find that face-centered-cubic (fcc) and body-centered-cubic (bcc) lattices exhibit different behaviors because of their different activation stresses of dislocation motions. Aside from $\sigma _{\rm y}$, the fracture behaviors and elastic moduli in broad $(D,l)$ space are also studied, which have not been explored before.

## RESULTS

### Generalization of HP and IHP behaviors

**Figure 1.**
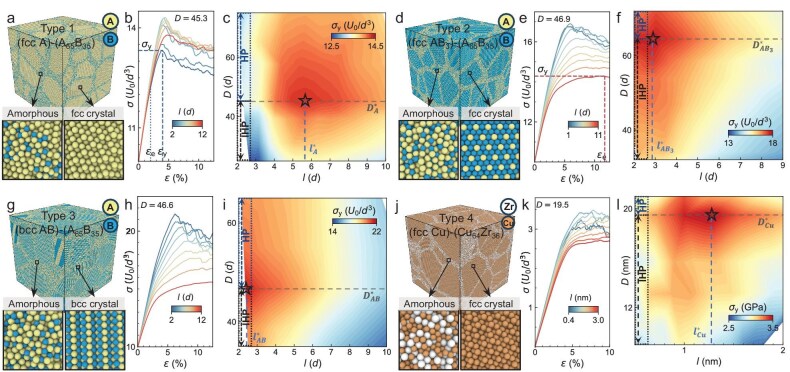
Four types of crystalline-amorphous composites, their corresponding engineering stress–strain curves, and generalized HP and IHP behaviors in $(D,l)$ space. (a) (fcc-A)-(A$_{65}$B$_{35}$) denotes 100% large atoms in fcc crystalline regions and a 65:35 mixture of large and small atoms in amorphous regions. (b) Stress–strain curves $\sigma (\varepsilon )$ of samples with a fixed $D=45.3$ and various $l$. Elastic limit $\varepsilon _{\rm e}$ represents the end of the linear regime. The maximum stress $\sigma _{\rm y}$ of each curve is used in (c). (c) Contour map of $\sigma _{\rm y}(D,l)$. The blue and black boxes at $l\approx 2$ are the conventional HP and IHP regimes, respectively. The horizontal dashed line represents the HP/IHP boundary at different $l$. The vertical dashed line represents the GB thickness $l^{*}$, with the maximum yield stress $\sigma _{\rm y}^{\rm max}$ labeled with an open star. Similar to (a–c), the results of the three other types of systems are shown in (d–f) for (fcc-AB$_{3}$)-(A$_{65}$B$_{35}$), (g–i) for (bcc-AB)-(A$_{65}$B$_{35}$) and (j–l) for (fcc-Cu)-(Cu$_{64}$Zr$_{36}$) composites.

We systematically vary $D$ and $l$ in four types of systems (Fig. 1a, d, g and j) and measure their $\sigma _{\rm y}(D,l)$ by molecular dynamics simulations. Type-1–3 systems are composed of binary-sized atoms, with the Lennard–Jones (LJ) pair potential $U(r)=4U_0[({d}/{r})^{12}-({d}/{r})^6]$ shifted to zero at $r>r_\textrm {c}=2.5d$, where $d$ is the length unit. For large (A-type) atoms, $d_{\rm AA}=1$; for small (B-type) atoms, $d_{\rm BB}=0.88$; and $d_{\rm AB}/d_{\rm AA}=0.8$ following the Kob–Anderson binary LJ mixture model [25]. In amorphous GBs, the number ratio between A and B atoms is set as 65:35 because large A and small B atoms can mix well without phase separation [26]. In crystalline grains, they are set as 100:0, 25:75 and 50:50 for type-1–3 systems, respectively. Type-4 systems comprise pure Cu in crystalline grains and a 64:36 mixture of Cu and Zr atoms in GBs. All the number ratios above commonly exist in real alloys [27]. The Cu-Zr mixture is a prototype of metallic glass [28]. The atomic interactions are described by embedded-atom-method potentials [29]. Crystalline regions are fcc for type-1, type-2 and type-4 systems (Fig. 1a, d and j) and bcc for type-3 systems (Fig. 1g). Uniaxial compression with a total strain of $\varepsilon =12.5\%$ and $\varepsilon =10\%$ is applied on LJ systems and Cu-Zr systems, respectively, all of which exceed their yielding points. Additional simulation details are given in Section S1.

The stress–strain curves $\sigma (\varepsilon )$ around the HP-IHP crossover (Fig. 1b, e, h and k) are averaged over three samples compressed along the $x$, $y$ and $z$ directions for sufficient statistics. Both the maximum stress (i.e. yield stress $\sigma _{\rm y}$) and flow stress $\sigma _{\rm f}$ given by the plateau height of $\sigma (\varepsilon )$ have been commonly used as the material strength, and they exhibit similar HP/IHP behaviors [8]. However, some samples exhibit strain softening without a plateau in $\sigma (\varepsilon )$. Thus, we show HP/IHP behaviors in terms of $\sigma _{\rm y}$ in the main text and in terms of the ambiguous $\sigma _{\rm f}$ in Fig. S5.

The contour maps of $\sigma _{\rm y}(D,l)$ (Fig. 1c, f, i and l) can be viewed as the generalization of the HP and IHP behaviors of $\sigma _{\rm y}(D)$. Conventional HP and IHP behaviors of $\sigma _{\rm y}(D)$ are well reproduced in thin-GB polycrystals ($l\approx 2$). The presence of triple junctions increases $l$ to about 2 for polycrystals. The maximum of $\sigma _{\rm y}(D,l\!=\!2)$ is observed at $D^{*}\approx 50$ atoms (Fig. 1c, f, i and l), which agrees well with the conventional HP-IHP crossover regimes of atomic polycrystals [8]. Diameter $D^{*}$ remains at about 50 atoms on the generalized HP-IHP boundary under different $l$ values (horizontal dashed lines in Fig. 1c, f, i and l). The maxima of $\sigma (D)$ at various $l$ form a ridge along the $l$ axis in Fig. 1c, f, i and l, indicating that they are not sensitive to $l$ and the Hall–Petch regime maintains a constant at different $l$. Similar to $\sigma _{\rm y}(D)$, $\sigma _{\rm y}(l)$ is also non-monotonic for type-1 and type-4 systems. The maximum $\sigma _{\rm y}$ is at $(D^{*},l^{*})=(45.3,5.8)$ for type-1 systems (denoted with an open star in Fig. 1c) and at $(D^{*},l^{*})=(19.5,1.5)\, \textrm {nm}=(60,5)$ Cu atoms for type-4 systems (denoted with an open star in Fig. 1l), indicating that $(D^{*},l^{*})$ slightly depends on atom interaction.

The deformations of polycrystals in conventional HP and IHP regimes occur via dislocation motions in crystalline grains and via deformations in GBs, respectively [5]. Small grains produce few dislocation pileups on GBs, thus requiring high applied stress to initiate plastic flow in crystalline grains; meanwhile, the presence of many GBs results in low required stress for GB deformation [6,8]. The competition between the required stresses for the two deformation mechanisms leads to non-monotonic $\sigma _{\rm y}(D)$ [5].

**Figure 2.**
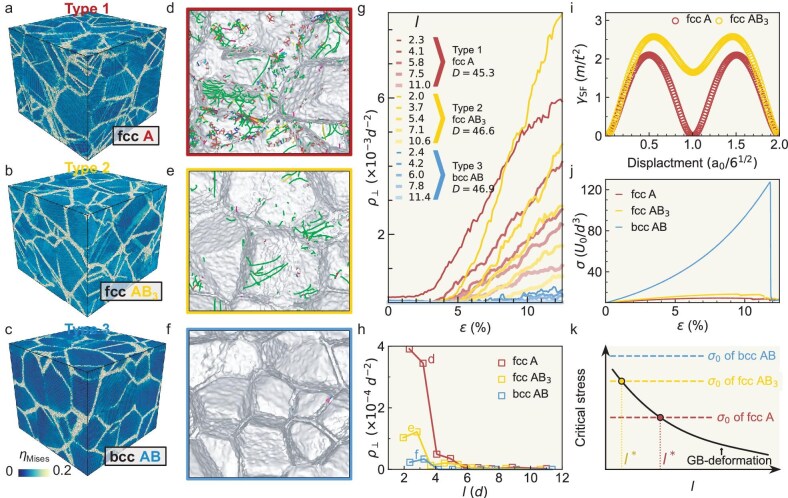
Lattice symmetry and composition effects on the plastic deformation mechanism of crystalline-amorphous composites. (a–c) Von Mises shear strain, $\eta _{_{\rm {Mises}}}$, of type-1–3 systems (Fig. 1a, d and g) under $\varepsilon =3.18\%$, $3.26\%$ and 4.50% at $(D,l)=(45.3,5.8)$, (46.9,2.8) and (46.6,2.4), respectively, corresponding to their $\sigma _{\rm y}^{\rm max}$ labeled with an open star in Fig. 1c, f and i. (d–f) Dislocation lines in the whole 3D samples of (a–c). Green, Shockley dislocation; pink, stair-rod dislocation; blue, full dislocation; red, other types of dislocations; grey, surfaces of the grains. They are all projected to a 2D plane. Defect formation processes in three dimensions are shown in Movies S1–S3. (g) Dislocation density $\rho _\perp$ in crystalline regions of type-1–3 systems during loading. The details of $\rho _\perp$ are given in Section S3.2. (h) Dislocation density $\rho _\perp$ at the midpoint between $\varepsilon _{\rm e}$ and $\varepsilon _{\rm y}$ in Fig. 1b, e, h and i. (i) Stacking fault energy $\\gtrsimmma _{\\small {sf}}$ as a function of the displacement between two adjacent $\lbrace 111\rbrace$ planes (Fig. S10b) [23] in type-1 and type-2 fcc single crystals. Here $a_{0}$ is the lattice constant. (j) Stress–strain curves of three defect-free single crystals with compositions A, AB and AB$_3$ under uniaxial compression along the [111] direction. The bcc crystal is much harder than fcc crystals and exhibits a sharp decrease at $\varepsilon =12\%$ (i.e. fracture). For these defect-free crystals, the fracture corresponds to the formation of the first dislocation [24]. (k) Schematic of the required stress $\sigma _0$ for plastic deformation in crystalline grains and GBs.

The above conventional mechanisms of the HP and IHP behaviors of $\sigma _{\rm y}(D)$ for polycrystals with $l\approx 2$ can similarly explain $\sigma _{\rm y}(l)$ in Fig. 1c, f, i and l. In the $l<l^{*}$ regime, thick GBs reduce the generation and motion of dislocations [17], leading to few dislocation pileups on GBs (Fig. 2 and Movies S1 and S2). Meanwhile, thick GBs act as a strong sink to absorb dislocations [30]. Both effects cause a low stress concentration on thick GBs, so high applied stress is required for plastic deformation. Such dislocation-motion-controlled deformation decreases (i.e. strength increases) as $l$ increases (Section S3.3). In the $l>l^{*}$ regime, GB deformations dominate plastic deformation (Fig. 2 and Movies S1 and S2), which agrees with previous simulations of crystalline-amorphous composites [20,22]. This increase in GB deformation (i.e. decrease in strength) as $l$ increases is consistent with the behaviors of glass nanopillars [31,32]. The above two types of deformation are further compared by their local deformations characterized by the von Mises shear strain $\eta _{_{\rm Mises}}$ (Equation S1). Atoms with $\eta _{_{\rm Mises}}>0.12$ are set as plastic deformation regions [33], which exist in grains by dislocation motions and in GBs by shear-transformation zones (Fig. 2a–c and Movie S1). Dislocation motions in crystalline grains dominate the deformation in the conventional HP regime, whereas GB deformations dominate in the conventional IHP regime [5]. We also compare the two types of deformations in the generalized $\sigma _{\rm y}(D,l)$ by measuring the number ratio of atoms that carry plastic deformations in GBs and in crystalline grains; see Section S3.1. The competition between dislocation-motion-controlled and GB-deformation-controlled strengths causes a peak in $\sigma _{\rm y}(l)$, as sketched in Fig. S11.

In contrast to the non-monotonic $\sigma _{\rm y}(l)$ at a fixed $D$ (Fig. 1c and l) for type-1 and type-4 systems, $\sigma _{\rm y}(l)$ decreases almost monotonically with the maxima at $(D^{*},l^{*})=(64.4, 2.8)$ for type-2 systems and at $(46.6, 2.4)$ for type-3 systems (Fig. 1f and i). This difference amongst systems indicates that $\sigma _{\rm y}(l)$ depends on crystal composition and lattice symmetry. We attribute the narrow regime of $l<l^{*}$ to the higher friction stress of moving dislocations in binary-composition fcc (type-2) and bcc (type-3) systems than in monodispersed-fcc (type-1 and type-4) systems. This mechanism is summarized in Fig. 2k. The minimum stress to deform the GB monotonically decreases with $l$ [9], whereas the minimum stress $\sigma _0$ required to move a dislocation should be independent of $l$ [34]. When the applied stress exceeds one of the two minimum required stresses above, one type of plastic deformation occurs and largely pre-empts the other type of deformation. Thus, the intersection of the two stresses in Fig. 2k gives $l^{*}$. Given that dislocations require high stress to move in bcc or binary crystalline grains ($\sigma _{\rm 0}^{\text{bcc-AB}}\gg \sigma _{\rm 0}^{\text{fcc-AB}_{3}}>\sigma _{\rm 0}^{\text{fcc-A}}$), their $l^{*}$ (Fig. 1f and i) is smaller than that of monodispersed fcc grains (Fig. 1e and l).

First, we compare type-1 and type-2 fcc systems. The dislocation lines (Fig. 2d and e) and dislocation densities $\rho _\perp$ (Fig. 2g and Fig. S9) show that numerous dislocations are generated in the type-1 system during yielding. The dislocation density $\rho _\perp$ decreases with $l$, indicating that thick GBs suppress the formation of dislocations. For type-1–3 systems, $\rho _\perp$ before reaching $\sigma _{\rm y}$ is nearly zero (Fig. 2h) for samples with $l\geqslant l^{*}$ in Fig. 1c, f and i, indicating that the plastic deformation is solely from GBs. Moreover, we observe that the dislocations in fcc systems are mainly 1/6$\\lesssimngle 112\rangle$ (Shockley) partial dislocations (Fig. 2d and e) that require lower stress to move compared with other types of dislocations [35]. They are often emitted from crystal-amorphous interfaces and propagate into crystalline regions (Movie S1). For fcc crystals, the minimum shear stress for creating partial dislocation, $\sigma _{\rm 0}$, linearly increases with the stacking fault energy $\\gtrsimmma _{\\small {sf}}$ [34]. We measure $\\gtrsimmma _{\\small {sf}}$ by the generated stacking fault method [23] (Fig. S10a and b) and obtain $\\gtrsimmma _{\\small {sf}}^{\text{fcc-AB}_{3}} >\\gtrsimmma _{\\small {sf}}^{\text{fcc-A}}$ (Fig. 2i). Thus, $\sigma _{\rm 0}^{\text{fcc-AB}_{3}} >\sigma _{\rm 0}^{\text{fcc-A}}$, and type-2 (fcc-AB$_3$) systems have fewer dislocations than type-1 (fcc-A) systems, which is in accordance with Fig. 2d and e and Movies S1 and S2. Moreover, unlike in type-1 systems whose dislocations appear immediately after the onset of plastic deformation (i.e. $\varepsilon >\varepsilon _{\rm e}$ in Fig. 1b), $\rho _\perp$ of type-2 systems is nearly zero (Fig. 2g) during the strain-hardening stage ($\varepsilon _{\rm e}<\varepsilon <\varepsilon _{\rm y}$ in Fig. 1e and Movie S2). Thus, dislocations barely contribute to the yield at $\varepsilon _{\rm y}$.

The bcc crystals have much higher friction stresses for dislocation motions than fcc crystals because of their low packing density, high activation energy of vacancy formation and asymmetric dislocation cores [35]. We compress fcc-A, fcc-AB$_3$ and bcc-AB single crystals and confirm that $\sigma _{\rm 0}^{\text{bcc-AB}} \gg \sigma _{\rm 0}^{\text{fcc-AB}_{3}}>\sigma _{\rm 0}^{\text{fcc-A}}$ (Fig. 2j). Thus, the dislocation density $\rho _\perp ^{\text{fcc-A}}>\rho _\perp ^{\text{fcc-AB}_{3}}\gg \rho _\perp ^{\text{bcc-AB}}$, which is in accordance with Fig. 2a–f, Movies S1–S3 and the literature [35].

### Temperature effect on $\sigma _{\rm y}(D,l)$

**Figure 3.**
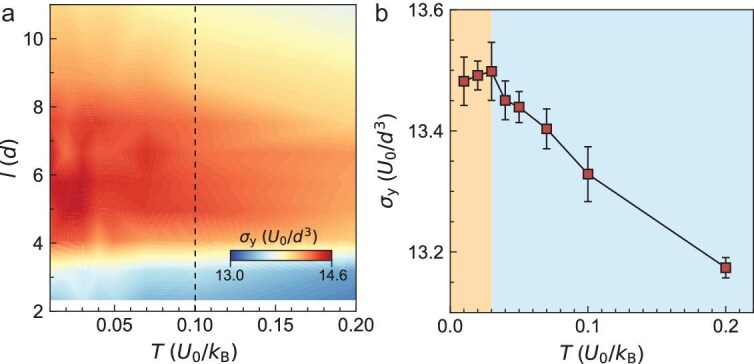
Temperature effect on yield stresses of type-1 systems. (a) The $\sigma _{\rm y}(l,T)$ of samples with $D=45.3$ and different $l$ (i.e. the horizontal dashed line in Fig. 1c for the HP-IHP boundary). Temperature $T$ is well below the melting temperature ($T_{\rm m} \approx 1.05$). We use $T=0.1$ (dashed line) in Figs 1, 2, 4. (b) The $\sigma _{\rm y}(T)$ of the polycrystal with $(D,l)=(45.3, 2.3)$ shows anomalous thermal strengthening (orange) and normal thermal softening (blue) regimes.

The conventional HP and IHP behaviors of $\sigma _{\rm y}(D)$ hold at different temperatures, and polycrystals are soft with low $\sigma _{\rm y}$ at high temperatures [8]. Here, we extend these temperature effects on $\sigma _{\rm y}$ to thick-GB composites for the first time. As temperature $T$ increases, $\sigma _{\rm y}(l)$ still peaks at $l^{*}=6$ for type-1 systems (Fig. 3a) and monotonically decreases for type-2 and type-3 systems (Fig. S7). Thus, the generalized HP and IHP behaviors of $\sigma _{\rm y}(D,l)$ hold similarly at different temperatures. The expected thermal softening is observed in the whole temperature range for type-1 systems with $l>4$ (Fig. 3a) and all type-2 and type-3 systems (Fig. S7). However, the $\sigma _{\rm y}(T)$ of type-1 systems with $l<4$ exhibits normal thermal softening at $T>0.03$ and anomalous strengthening at $T<0.03$ (Fig. 3b). This anomalous thermal strengthening was experimentally discovered very recently in pure metals at high strain rates and is attributed to the competition amongst thermal, athermal and drag strengthening mechanisms [36]. These complex competitions depend on lattice symmetry and composition, which may explain why anomalous thermal strengthening exists in type-1 polycrystals, but not in other systems.

### Fracture behaviors of composites with different $(D,l)$

Fracture behaviors determine if a solid is ductile or brittle. High ductility is desirable for engineering materials to prevent catastrophic failure during service [37]. Ductility can be enhanced by introducing twin boundaries, stacking faults or second-phase precipitates because they can block dislocation motions [38] and thus expand the strain-hardening regime and delay the onset of fracture [39,40]. However, introducing defects or precipitates usually weakens the material, which is undesirable [39]. Minimizing the strength-ductility trade-off is an important challenge in solid mechanics [41]. Expanding $l$ can enhance ductility because thick GBs act as high-capacity sinks for dislocations and thus expand the strain-hardening regime caused by deformations in crystalline grains [30]. Strength decreases with $l$ in most $(D,l)$ regimes (Fig. 1c, f, i and l), so the strength-ductility trade-off remains. However, $\sigma _{\rm y}(l)$ increases in $l<l^{*}$ (Fig. 1c and l), and thus the trade-off is absent in this regime.

Ductility and the related fracture behaviors of crystalline-amorphous composites have not been studied before. Here, we perform simulations on samples with free surfaces in the $x$ direction and with periodic boundary conditions in the $y$ and $z$ directions under tensile deformation along the $z$ direction. The abrupt decrease of $\sigma (\varepsilon )$ represents a fracture, that is, the appearance of a crack. Note that a fracture can also refer to the complete separation of the sample into two or more parts [42]. If no strong plastic deformation occurs before the fracture, the solid is brittle; otherwise, it is ductile [42]. Ductility can be characterized qualitatively by the fracture morphology and quantitatively by tensile strain $\varepsilon _{\rm f}$ at the fracture [38].

**Figure 4.**
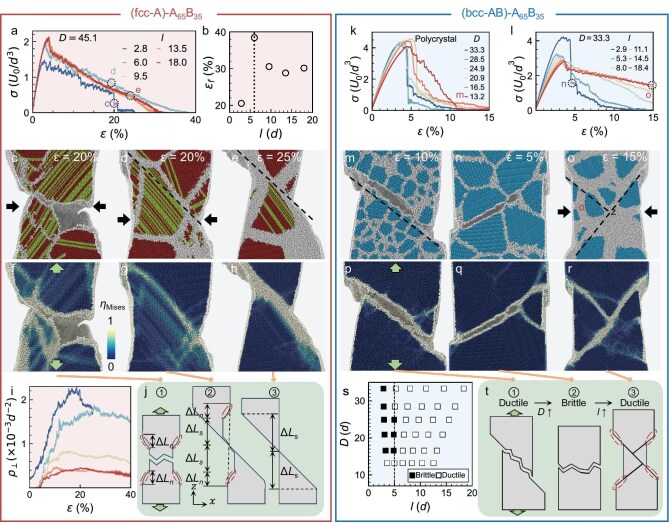
Fracture behaviors of type-1 systems with $D=45.1$ and type-3 systems. (a) Engineering stress–strain curves $\sigma (\varepsilon )$. (b) Fracture strain $\varepsilon _{\rm f}$ measured at $\sigma =0$ in (a) that reflects ductility. (c–h) Fracture morphologies of three samples with (c, f) $l=2.8$ at $\varepsilon =20\%$, (d, g) $l=6.0$ at $\varepsilon =20\%$ and (e, h) $l=18$ at $\varepsilon =25\%$. Each pair of black arrows in (c, d and o) represents a necking. The black dashed lines in (d, e, m and o) represent shear planes. Each atom is colored by its local lattice structure (red, fcc; green, hcp; blue, bcc; grey, amorphous) in (c–e) and von Mises shear strain in the corresponding (f–h). (i) Dislocation density in solids with various $l$; see the legend in (a). (j) Ductility mechanisms sketched by elongation $\Delta L_{\rm n,s}$: (1) necking (highlighted by ellipses) for (c, f), (2) necking followed by shear deformation in GBs for (d, g), and (3) shear deformation in GBs for (e, h). (k) The stress–strain curves $\sigma (\varepsilon )$ of type-3 polycrystals with $l\approx 2$. (l) The stress–strain curves $\sigma (\varepsilon )$ of type-3 composites with $D=33.3$ and $l>2$. (m–r) Fracture morphologies of three samples with (m, p) $(D,l)=(13.2,3.6)$ at $\varepsilon =10\%$, (n, q) $(D,l)=(33.3,2.9)$ at $\varepsilon =5\%$ and (o, r) $(D,l)=(33.3,18.4)$ at $\varepsilon =15\%$. Atoms in (m–o) and (p–r) are colored in the same way as in (c–e) and (f–h), respectively. (s) Ductile–brittle phase diagram of type-3 systems in $(D,l)$ space. (t) Schematic of ductility mechanisms: (1) single-plane shear deformation followed with a crack for (m, p), (2) crack along GBs for (n, q), and (3) necking induced by chisel edge separation via shear deformation along two perpendicular planes for (o, r). Full images of (c–h) and (m–r) are shown in Figs S13 and S14, respectively. The elongation processes of (c–h) and (m–r) and their $\sigma (\varepsilon )$ are shown in Movies S4–S9.

Type-1 fcc systems are ductile because their stress–strain curves in Fig. 4a decrease after the peak and reach 0 at large $\varepsilon _{\rm f}$. The tensile strain $\varepsilon _{\rm f}(l)$ peaks at $l=6$ (Fig. 4b). We attribute this maximum ductility at $l=6$ to the fact that the sample has both types of deformation, whereas small- and large-$l$ samples only have one type of deformation (Fig. 4j). In small-$l$ samples ($l<6$; e.g. Fig. 4c and f), the plastic elongation along the $z$ direction is $2\Delta L_{\rm n}$ because of necking (mechanism (1) in Fig. 4j). Necking is a type of plastic deformation with a prominent decrease in the local cross-sectional area, (Fig. 4c and mechanism (1) in Fig. 4j). Necking arises from deformations via considerable dislocation motions and twin-boundary generations in crystalline grains [42]. This result is confirmed by the proliferation of hexagonal close-packed (hcp) lattices (green regions in Fig. 4c–e) that can be viewed as twin boundaries in fcc lattices. At the end of the necking process, a crack initiates on a free surface and then propagates along the shortest GBs that cross the two free surfaces (Fig. S13a and b and Movie S4). In large-$l$ samples ($l>6$), many dislocations are absorbed into GBs (Fig. 4i), making the crystalline grains less stretchable. Thus, deformations within GBs pre-empt deformations in crystalline grains (i.e. necking). Plastic deformations mostly occur via shear deformation within thick GBs (Fig. 4e and h, and Fig. S13e and f and Movie S6), which produce elongation $2\Delta L_{\rm s}$ (mechanism (3) in Fig. 4j). The 45$^\circ$ shear plane relative to the tensile elongation direction endures the maximum shear stress [43], so the shear deformation is along the GBs whose orientations are close to 45$^\circ$ (dashed lines in Fig. 4d and e). As depicted in Fig. 4j, the relative contribution of $\Delta L_{\rm s}$ increases with $l$ (i.e. the relative contribution of $\Delta L_{\rm n}$ decreases with $l$) because the shear plane forms more easily in thicker GBs. In the samples with $l=6$, both the necking at the early stage and the 45$^\circ$ shear deformation at the late stage (Fig. 4d and g, and Fig. S13c and d and Movie S5) contribute to the elongation. Thus, the total elongation is $2(\Delta L_{\rm s}+\Delta L_{\rm n})$ (mechanism (2) in Fig. 4j), which is greater than those of samples with $l<6$ and $l>6$. This explains the maximum ductility at $l=6$ (Fig. 4b). Whether the occurrence of the maximum ductility and strength at $l=6$ is a coincidence deserves future studies.

Unlike the type-1 fcc samples that are all ductile (Fig. 4a), the type-3 bcc samples with different $(D,l)$ can be ductile with large $\varepsilon _{\rm f}$ or brittle with an abrupt decrease in $\sigma (\varepsilon )$ right after the peak (Fig. 4k, l and s). For brittle samples in Fig. 4k and l, although $\sigma$ is non-zero after the abrupt drop, a large crack has already formed (Movie S7), indicating that a fracture has occurred. The brittle and ductile behaviors of the bcc samples are summarized in Fig. 4s, with the ductile–brittle transitions occurring at $l\approx 6$ for $D >15$ and at $l<6$ for $D\approx 15$. Ductile–brittle transitions occur in many polycrystals by increasing the grain size $D$ [44–46]. Figure 4s not only confirms the conventional $D$ effect, but also shows the $l$ effect on the ductile–brittle transition.

The fracture morphology is characterized by a crack along the GBs with a rough surface for brittle solids (Fig. 4n and q, mechanism (2) in Fig. 4t, and Fig. S14c and d and Movie S8) and shear deformation with a rough (Fig. 4m and p, mechanism (1) in Fig. 4t, and Fig. S14a and b and Movie S7) or smooth (Fig. 4o and r, mechanism (3) in Fig. 4t, and Fig. S14e and f and Movie S9) surface for ductile solids. The crack initiates from a free surface in the ductile samples and from a triple junction inside the bulk in the brittle samples. When the crack on the shear band produces voids (Fig. S14a and b), the subsequent fracture surface becomes rough [42]. A void-free fracture exhibits a smooth surface (e.g. Fig. 4e, h, o and r). Ductile deformation occurs via a single 45$^\circ$ shear plane along GBs for small-$D$ polycrystals (Fig. 4m, p and mechanism (1) in Fig. 4t) and via two shear planes with $\pm 45^\circ$ for large-$l$ composites (Fig. 4o, r and mechanism (3) in Fig. 4t).

### Elastic properties in $(D,l)$ space

Elastic moduli are baseline properties of solids and are closely related to the energy barrier for yielding and flow, but they have not been explored in the $(D,l)$ space. We measure the elastic stiffness tensor $C_{ij}$ and obtain the bulk modulus $K=(C_{11}+2C_{12})/3$, shear moduli $G_1=C_{44}$ and $G_2=(C_{11}-C_{12})/2$, and Young’s modulus $E=2C_{44}(C_{11} + 2C_{12})/(C_{11} + C_{12})$. The two shear moduli are very close (Fig. S3), indicating that the sample contains a sufficient number of grains to be regarded as isotropic. The Young’s moduli and bulk moduli in the $(D,l)$ space are shown in Fig. S15.

**Figure 5.**
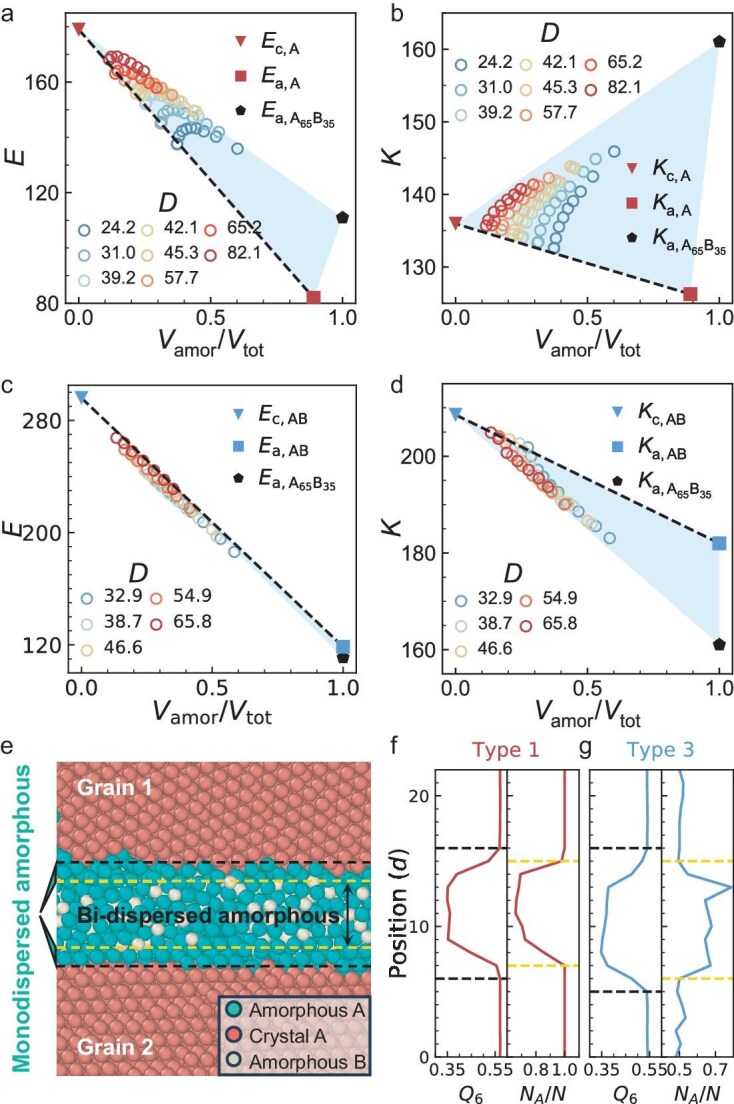
Elastic moduli of type-1 systems and type-3 systems. (a, c) Young’s modulus $E$ and (b, d) the bulk modulus $K$. All moduli fall in the blue triangular area, which is delimited by the moduli of the single crystal (triangle), purely amorphous glass (square) and interfacial phase with an amorphous structure and crystal composition (pentagon). For the red squares in (a, b), $V_{\rm amor}/V_{\rm tot}<100\%$ because small crystallites inevitably exist in the amorphous sample composed of monodispersed atoms. (e) The crystalline-amorphous interface (black dashed lines) and monodispersed-bidispersed interface (yellow dashed lines) are off by one layer of atoms, resulting in two monodispersed amorphous interfacial layers. (f, g) Profiles of the mean crystalline order ($Q_6$) and fraction of large atoms ($N_{\rm A}/N$) averaged along the interface in (f) type-1 and (g) type-3 systems. Parameter $Q_6$ and $N_A/N$ rapidly change at slightly different positions (black and yellow dashed lines, respectively).

For polycrystals, the elastic modulus is a linear summation of the crystalline and amorphous parts weighted by their volume fractions, following the simple rule of mixture: $E=\sum _{i=1}^{n}E_{i}f_{i}$ [47] with $E_{i}$ the elastic modulus and $f_{i}$ the volume fraction of the $i$th phase. This linear relationship is confirmed in our polycrystal samples: the data point with the lowest amorphous fraction at each $D$, i.e. the polycrystal with the lowest $l$, lies on the dashed line between the triangle (pure crystal) and square (pure amorphous) in Fig. 5a–d. However, the other data points deviate from the dashed line in Fig. 5a, b and d. To restore the rule of mixtures, we find that the crystalline-amorphous interfaces need to be regarded as the third phase because the interfacial layers have amorphous structures but the same composition as crystalline regions. The crystalline order can be characterized by the bond-orientational order parameter $Q_{6}$ (Equation S2) [48]. Parameter $Q_{6}$ and the composition change sharply in the normal direction of GBs (Fig. 5f and g), indicating sharp crystalline-amorphous and atom composition interfaces. However, the two interfaces are offset by one layer of atoms, resulting in one layer of the third phase (Fig. 5f and g). The moduli of the three pure phases measured in separate simulations form a blue triangular area in Fig. 5a–d that covers all the data points, and each data point is a linear combination of these three phases following the rule of mixtures.

## DISCUSSION

We measure the stress–strain relation $\sigma (\varepsilon )$ (e.g. Fig. 1b, e, h and k) for four types of samples (Fig. 1a, d, g and j) with various $(D,l)$ and study the initial elastic regime at small strains (elastic moduli in Fig. 5), the plastic regime at medium strains (for the HP and IHP behaviors of $\sigma _{\rm y}$, see Fig. 1c, f, i and l; for their mechanisms, see Fig. 2) and fracture behaviors at large strains (Fig. 4). These behaviors have been studied as a function of $D$ [5–8,28,37,47], but not as a function of $(D,l)$.

The HP and IHP behaviors are generalized from $\sigma _{\rm y}(D)$ to $\sigma _{\rm y}(D,l)$. Under a fixed $l$, $\sigma _{\rm y}(D)$ is non-monotonic (i.e. with HP and IHP behaviors), with the maximum at a fixed $D\approx 50$ atoms for different $l$. Under a fixed $D$, $\sigma _{\rm y}(l)$ is non-monotonic for fcc composites and monotonic for bcc composites. The maximum strength $\sigma _{\rm y}^{\rm max}$ is at $(D,l)\approx (50,6)$ atoms for fcc composites with monodispersed crystalline regions, and at $(D,l)\approx (50,2)$ atoms for fcc composites with bidispersed crystalline regions and bcc compositions (Fig. 1c, f, i and l). These results can explain the recent experimental results on crystalline-amorphous composites. For example, the maximum strength of conventional polycrystals can be exceeded in fcc Ni-Mo crystalline-amorphous composites with 3-nm-thick (i.e. around 10 Mo atoms) GBs [17], but cannot be exceeded in the bcc Co-Al alloys with 2–10-nm-thick GBs [18]. We suggest that reducing $l$ to 2 nm (i.e. $l^{*}=6$ atoms) in fcc Ni-Mo composites can further enhance strength.

Increasing $l$ and decreasing $D$ have similar effects on $\sigma _{\rm y}$ because they both increase the volume fraction of amorphous structures. However, $\sigma _{\rm y}$ is not solely controlled by the amorphous fraction (Fig. S4). The maximum $\sigma _{\rm y}$ is at an amorphous fraction of 28% in type-1 systems and 15% in type-2–4 systems (Fig. S4). At large $D$ and small $l$, plastic deformations primarily arise from dislocation motions inside grains. At small $D$ or large $l$, i.e. a high amorphous fraction, plastic deformations mainly occur in GBs via shear deformation (Fig. 2). Aside from these common features shared by all systems, $\sigma _{\rm y}(l)$ peaks at $l=6$ for fcc composites and monotonically decreases for bcc composites, which is explained as follows. Expanding $l$ up to six atoms can reduce dislocation motions and thus enhance $\sigma _{\rm y}$ in fcc composites, but it is not effective in bcc composites because dislocations barely move due to their high friction stress (Fig. 2g). The sample formation conditions (e.g. cooling rate, relaxation time) can affect the properties of the amorphous regions[49]. For example, a longer annealing time shifts $\sigma _y(l)$ to a higher strength, but barely affects the peak position $l^{*}$. Further discussion is provided in Section S2.3.

The temperature dependence of $\sigma _{\rm y}(l)$ (Fig. 3a) shows that conventional thermal softening in polycrystals also exists in crystalline-amorphous composites, and reveals an abnormal increasing $\sigma _{\rm y}(T)$ regime at low $T$ (Fig. 3b). This abnormal regime was discovered recently in polycrystalline metals [36].

For fcc composites, increasing $l$ up to six atoms enhances strength (Fig. 1c) and ductility (Fig. 4b), so the strength-ductility trade-off is avoided. Ductility mainly arises from necking caused by grain deformation in small-$l$ samples and from shear deformation in GBs in large-$l$ samples. Both deformation mechanisms exist in intermediate-$l$ samples, resulting in the maximum ductility at $l=6$ (Fig. 4b and j). For bcc composites, however, increasing $l$ improves ductility, but entails the trade-off of reduced strength. All fcc composites are ductile, and bcc composites exhibit a ductile–brittle transition at $l=6$ for $D>15$ samples and at $l<6$ for $D=15$ samples (Fig. 4s).

Additionally, we find that the rule of mixtures for elastic moduli holds well for thick-GB composites. However, when crystalline and amorphous regions have different compositions, their interfaces should be regarded as the third phase to recover the rule of mixtures (Fig. 5).

The results show that the poorly explored structural parameter $l$ has similar importance for mechanical properties as $D$. The results for $(D,l)$ space can guide the fabrication of ultrastrong solids. Experimentally testing these simulation results is feasible because thick-GB composites can be fabricated in some alloys [13,16–18], glass-ceramics [11] and polymer crystals [50]. Currently, the primary methods for fabricating crystalline-amorphous composites include solute segregation, defect introduction into confined regions via annealing and compression or deposition after magnetron sputtering [15,51,52]. In addition, $(D,l)$ can be tuned by varying chemical compositions under different pressures [51,52]. While some alloys have attained a few values near the optimal $(D^{*},l^{*})$ regime[15], precise control over $D$ and $l$ remains challenging. Future efforts could systematically explore this parameter space by fabricating numerous samples with random $D$ and $l$ values, potentially covering the optimal regime. Additionally, high-throughput alloy fabrication methods [53] enable rapid exploration of a broad parameter space. Combining such approaches with controlled solute concentrations, annealing conditions or sputtering duration may enable better control over $(D,l)$ near the optimal regime. Besides the strength and elastic moduli, other mechanical properties such as ductility, fracture and tension-compression asymmetries can be similarly studied in the broad $(D,l)$ space. In addition, tuning $(D,l)$ provides a new platform to study the ductile–brittle transition and the poorly explored polycrystalline-amorphous transition [54].

## Supplementary Material

nwaf336_Supplemental_Files
