# Supplementary material for: Mechanical properties of crystalline-amorphous composites: generalization of Hall–Petch and inverse Hall–Petch behaviors
Source: Natl Sci Rev. 2025 Aug 20;12(9):nwaf336. doi: 10.1093/nsr/nwaf336 (PMC12421585; doi:10.1093/nsr/nwaf336)
Supplement: nwaf336_Supplemental_Files [file nwaf336_supplemental_files.zip › Supplementary data.pdf]

Supplementary Data

Mechanical properties of crystalline-amorphous composites: generalization of Hall-Petch and inverse Hall-Petch behaviors

Zhibin Xu, Mengmeng Li, and Yilong Han[*]

Department of Physics, The Hong Kong University of Science and Technology, Clear Water Bay, Hong Kong, China

August 2, 2025

[*]Corresponding authors. email:yilong@ust.hk

Contents

1 Method **3**

1.1 Construction of polycrystals with thick grain boundaries (GBs) 3

1.2 Sample characterizations 4

1.3 Sample isotropy 5

2 Generalized HP and IHP behaviors **6**

2.1 Amorphous fractions on the contour plots of $\sigma_{\mathrm{y}}(D, l)$ 6

2.2 Using flow stress as strength 6

2.3 Effects of strain rate and relaxation time on $\sigma_{\mathrm{y}}(D, l)$ 6

2.4 Temperature effect on $\sigma_{\mathrm{y}}(D, l)$ 8

3 Mechanisms of the generalized HP and IHP behaviors **9**

3.1 Ratio of deformations in GBs and in crystalline grains 9

3.2 Dislocation density during deformation 10

3.3 Required stress to create a dislocation in different crystals 10

3.4 Plastic deformations are more uniform in thicker GBs 11

4 Fracture process **12**

5 Elastic Moduli **15**

6 Movies **16**

1 Method

1.1 Construction of polycrystals with thick grain boundaries (GBs)

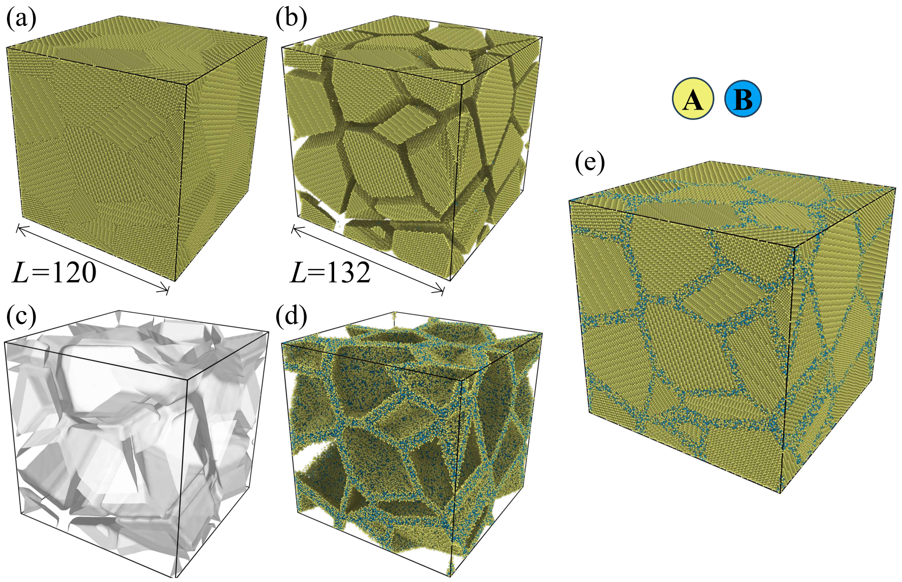

Figure S1: Procedure of constructing a crystalline–amorphous composite from a polycrystal. (a) Initial binary polycrystal with mean grain diameter $D = 46.57$ in a cubic box with side length $L = 120$. (b) Shifted grains in the expanded cubic box with $L = 132$. (c) Crystalline–amorphous interfaces in (b). (d) In an amorphous solid composed of $A_{65}B_{35}$ mixture of Lennard–Jones atoms, the atoms in the region given by (c) are chosen. (e) Final sample with thick GBs obtained by combining (b) and (d).

In previous simulations, crystalline–amorphous composites were constructed by quenching liquids [1], substituting surface layers of crystalline grains to amorphous structures [2] or using periodic grain patterns [3]. These methods do not produce a series of samples with the same crystalline regions and different GB thicknesses, or the grains are too regular to mimic real materials. Here, we propose a method to create the desired GB thickness and maintain the same sizes and irregular shapes of crystalline grains so that the GB thickness effect can be well measured by comparing a series of samples. As illustrated in Figure S1, the crystalline–amorphous composite is constructed through the following steps. Firstly, a polycrystal is generated using the Voronoi tessellation method [4] (Figure S1a). The shape of each grain is given by the Voronoi cell. The lattice orientations of the grains are set as random. Secondly, the simulation box is expanded, and the center of mass of each grain is proportionally projected into the new box. Such a shift of grains creates gaps with uniform thickness l between grains (Figure S1b). Thirdly, surface meshes are created by α-shape method [5] to describe the shape of gaps (Figure S1c). Fourthly, the surface meshes are used to extract thick-GB regions from an amorphous glass (Figure S1d). This glass is created by rapidly cooling a liquid from $3.5T^*$ to $0.1T^*$ within 100τ under $10P^*$. Last, the displaced grains in Figure S1b are combined with the GBs in Figure S1d to produce a thick-GB sample (Figure S1e). The number of atoms ranges from 2.5 million to 11.4 million for different samples. The structures in Figure 1a,d,g,j, Figure 2a–c, Figure 3c of the main text and Figures S1, S8, and S11a are visualized using the OVITO visualization tool [6].

The initial polycrystals are in the same-sized cubic box ($L = 120d$), so a polycrystal with large-D grains contains a small number of grains (Figure S1). The diameter of the large particle, d, is used as the length unit. After GBs with different thicknesses are inserted, the box sizes become slightly different. (D, l) are tuned in four types of systems (Figure 1a,d,g,j of the main text). (1) The face-center-cubic (fcc) crystalline grains composed of large (A-type) atoms and amorphous GBs composed of large and small (B-type) atoms with a number ratio of $N_A : N_B = 65 : 35$, i.e. (fcc-A)-($A_{65}B_{35}$). The number ratio $N_A : N_B = 65 : 35$ can form stable amorphous structures without phase separation [7],

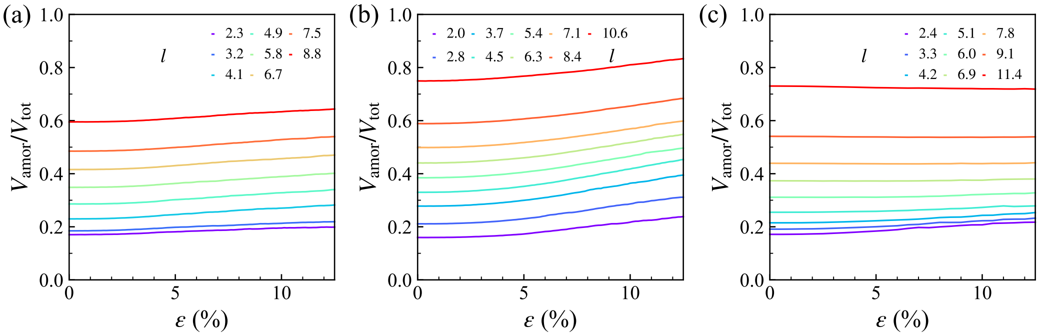

Figure S2: Fraction of the amorphous phase under loading strain ε. (a) Type 1 systems with D = 45.3. (b) Type 2 systems with D = 46.9. (c) Type 3 systems with D = 46.6.

so it is used in all amorphous regions. (2) The second type is a binary solid with $N_{\text{A}} : N_{\text{B}} = 1 : 3$ in fcc crystalline grains and $N_{\text{A}} : N_{\text{B}} = 65 : 35$ in amorphous GBs, i.e. (fcc-AB_3)-($\text{A}_{65}\text{B}_{35}$). (3) The third type is a binary solid with $N_{\text{A}} : N_{\text{B}} = 1 : 1$ in the body-centered-cubic (bcc) crystalline grains and $N_{\text{A}} : N_{\text{B}} = 65 : 35$ in amorphous GBs, i.e., (bcc-AB)-($\text{A}_{65}\text{B}_{35}$). (4) The fourth type refers to fcc crystalline grains composed of copper (Cu) atoms and amorphous GBs composed of a copper–zirconium (Cu–Zr) mixture with $N_{\text{Cu}} : N_{\text{Zr}} = 64 : 36$, i.e. (fcc-Cu)-($\text{Cu}_{64}\text{Zr}_{36}$). After relaxation, all crystalline regions are barely amorphized during the deformation process (Figure S2).

All simulations are performed with the Large-scale Atomic/Molecular Massively Parallel Simulator (LAMMPS) [8]. Types 1–3 systems are composed of atoms with the Lennard–Jones (LJ) potential. The interactions between binary atoms are set to follow the Kob–Anderson mixture [9]: $d_{\text{AA}} = 1.0$, $d_{\text{BB}}/d_{\text{AA}} = 0.88$ and $d_{\text{AB}}/d_{\text{AA}} = 0.8$; $U_{\text{AA}} = 1.0$, $U_{\text{BB}}/U_{\text{AA}} = 0.5$ and $U_{\text{AB}}/U_{\text{AA}} = 1.5$; all masses are set to $m = 1.0$. Pressure, time and temperature have units $P^* = U_0/d^3$, $\tau = \sqrt{md^3/U_0}$ and $T^* = U_0/k_{\text{B}}$, respectively. Boltzmann constant $k_{\text{B}} = 1$. The Types 1–3 samples are relaxed for 700τ at $0.1T^*$ and $10P^*$. Type 4 systems are composed of Cu and Zr atoms with multibody embedded-atom-method potentials [10]. Type 4 samples are relaxed at $T = 300$ K and $P = 0$ for 1 ns. Uniaxial compression is applied on Types 1–3 LJ samples with strain rate $\dot{\varepsilon} = 2.5 \times 10^{-4} t^{-1}$ and Type 4 Cu–Zr samples with $\dot{\varepsilon} = 2.5 \times 10^{8} \text{s}^{-1}$.

1.2 Sample characterizations

Common neighbor analysis (CNA) [11] is implemented to identify each particle's local lattice symmetry. Thick GBs occasionally contain a few tiny ($D < 3$) crystallites that are not counted as grains. Mean grain diameter $D \equiv (\sum_i \sqrt[3]{6V_i/\pi})/N_i$, where V_i and N_i are the volume and number of atoms of grain i, respectively. GB thickness $l \equiv 2V_{\text{GB}}/A_{\text{c}}$, where V_{GB} is the total volume of the thick amorphous GBs and A_{c} is the total surface area of all crystalline grains. The presence of triple junctions increases the mean GB thickness, so the measured $l \approx 2$ instead of 1 in polycrystals.

Von Mises shear strain is commonly used to characterize local shear strain [12]. To visualize the deformation mechanism, we measure the von Mises shear strain as follow:

$$\eta_{\text{Mises}} = \sqrt{\eta_{\text{xy}}^2 + \eta_{\text{yz}}^2 + \eta_{\text{zx}}^2 + \frac{(\eta_{\text{xx}} - \eta_{\text{yy}})^2 + (\eta_{\text{yy}} - \eta_{\text{zz}})^2 + (\eta_{\text{zz}} - \eta_{\text{xx}})^2}{6}}. \tag{1}$$

η_{ij} is the Green-Lagrangian strain tensor calculated by $\eta_{ij} = (\mathbf{F}^{\text{T}}\mathbf{F} - \mathbf{I})/2$ [13], where $\mathbf{F}$ is the deformation gradient tensor. $\mathbf{I}$ is the 3×3 identity tensor, with ones on the diagonal and zeros elsewhere.

We use the explicit deformation method to measure elastic stiffness tensor C_{ij} because it has better accuracy [14]. On the basis of C_{ij}, we calculate bulk modulus $K = (C_{11} + 2C_{12})/3$ and Young's modulus $E = 2C_{44}(C_{11} + 2C_{12})/(C_{11} + C_{12})$.

The local crystalline order of particle i is characterized by its bond-orientational order parameter:

$$Q_{i,\iota} = \sqrt{\frac{4\pi}{2\iota+1}\sum_{m=-\iota}^{\iota}\left|\sum_{j=1}^{n}\frac{A_j}{A}Y_{\iota m}\left(\theta_j,\phi_j\right)\right|^2}, \tag{2}$$

where θ_j and ϕ_j are spherical polar angles of the bond vector from particle i to its jth neighbor, n is the number of nearest neighbors of particle i, A_j is the area of the Voronoi facet to the jth neighbor. A is the total surface area of the Voronoi cell and $Y_{\iota m}$ is a spherical harmonic function of degree ι and order m. We use six-fold ($\iota = 6$) bond-orientational order parameter Q_6 to distinguish crystalline and amorphous phases.

1.3 Sample isotropy

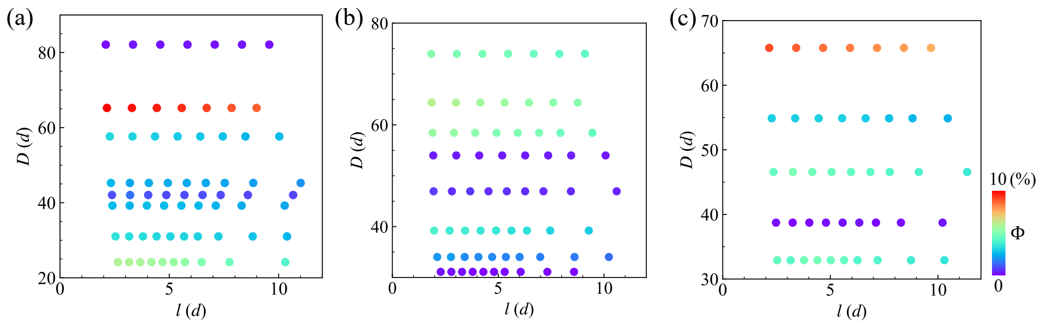

Figure S3: Degree of sample anisotropy Φ for (a) Type 1, (b) Type 2, and (c) Type 3 systems. $\Phi = 0$ means that the sample is fully isotropic.

When grains are large, the sample contains a small number of grains, which makes the sample not fully isotropic. The degree of anisotropy is characterized by the modified Zener ratio [15] $\Phi = |G_1 - G_2|/(G_1 + G_2)$, where $G_1 = (C_{11} - C_{12})/2$ and $G_2 = C_{44}$ are the two shear moduli. $\Phi_{\text{iso}} < 5\%$ for most samples, and the maximum is about 10% (Figure S3). Thus the samples are quite isotropic. Moreover, each data point of Φ_{iso} in Figure S3 is from a single measurement, and we minimize the effect of anisotropy by averaging the results of uniaxial compression in x, y, and z directions.

2 Generalized HP and IHP behaviors

2.1 Amorphous fractions on the contour plots of $\sigma_\mathrm{y}(D, l)$

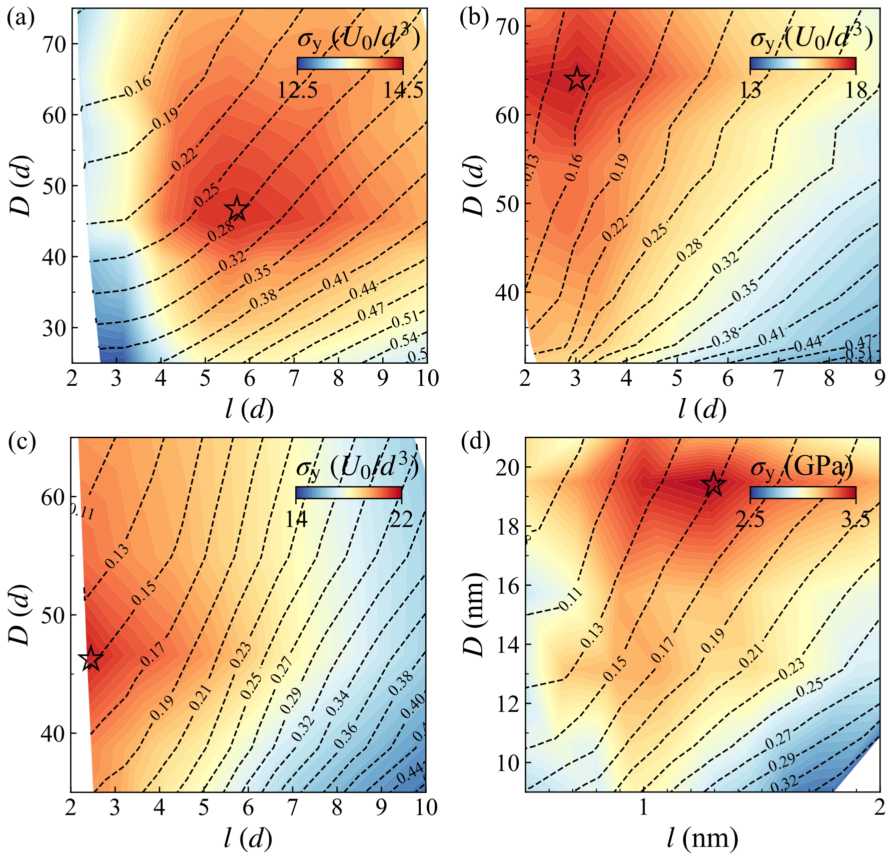

Figure S4: Contour maps of amorphous volume fraction ϕ_amor (black curves) overlaid on contour maps of σ_y for systems 1–4 in Figure 1c,f,i,l of the main text. $\sigma_\mathrm{y}^\mathrm{max}$ labeled with ☆ is at ϕ_amor = 28% in Type 1 systems and at ϕ_amor = 15% in Types 2–4 systems.

The ratio of the volume of amorphous regions to the volume of the whole sample, ϕ_amor, is labeled on $\sigma_\mathrm{y}(D, l)$ shown in Figure S4. When ϕ_amor is fixed, $\sigma_\mathrm{y}(l)$ and $\sigma_\mathrm{y}(D)$ are usually non-monotonic, and the maximum σ_y is achieved at different (D, l) under different ϕ_amor (Figure S4).

2.2 Using flow stress as strength

The strength of a solid can be defined as the maximum stress (i.e. yield stress σ_y) [1] or flow stress [16]. Both definitions are commonly used in HP/IHP behaviors. The stress–strain curves of some samples decrease continuously without a plateau (e.g. Figure 1a,b,c of the main text), so flow stress is not rigorously defined. Nevertheless, we define σ_f as the average stress in $0.1 < \varepsilon < 0.125$ for Types 1–3 systems and as the average stress in $0.08 < \varepsilon < 0.1$ for Type 4 systems (i.e. the plateau regimes in Figure 1b,e,h,k of the main text). σ_y and σ_f are measured by averaging the results of the uniaxial compressions along x, y, and z directions in the samples with different initial thermal motions of atoms for sufficient statistics. Their contour plots are shown in Figure 1c,f,i,l of the main text for σ_y and in Figure S5 for σ_f.

2.3 Effects of strain rate and relaxation time on $\sigma_\mathrm{y}(D, l)$

Our strain rate $\dot{\varepsilon}$ is comparable to those used in all recent simulations about the deformation of crystal–amorphous composites (e.g., refs. [2, 3]) and a few laser-induced impact experiments [17, 18].

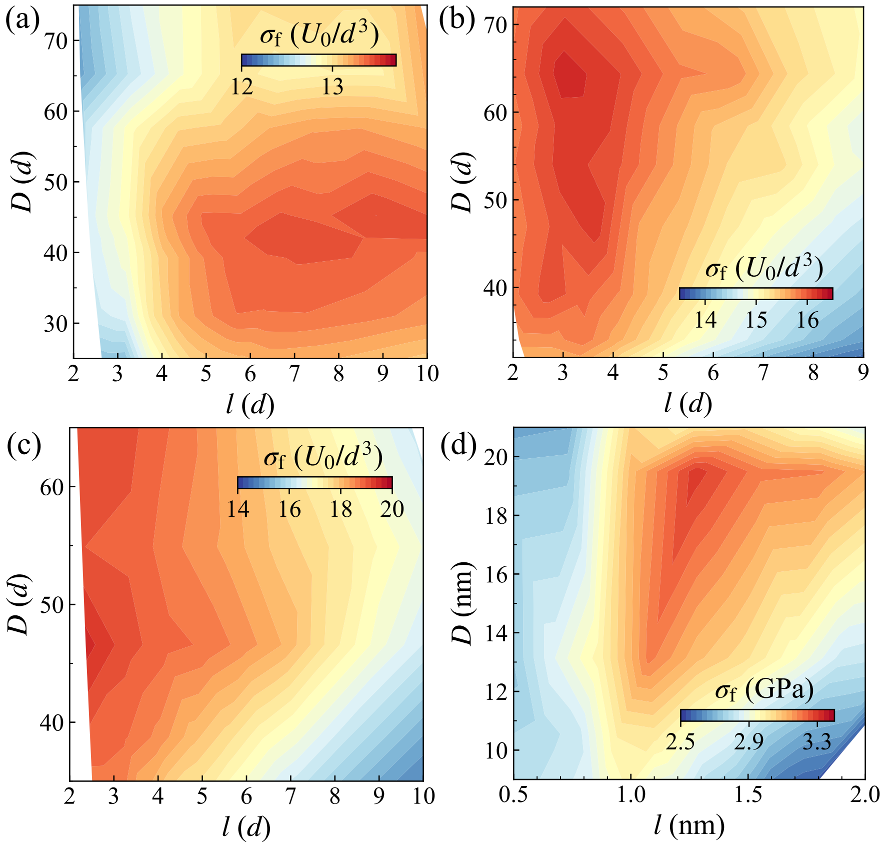

Figure S5: Contour maps of flow stress $\sigma_f(D, l)$ for samples with different mean grain diameters D and GB thicknesses l. d is the diameter of large atoms. (a) Type 1 (fcc A)-($A_{65}B_{35}$) systems. (b) Type 2 (fcc-AB_3)-($A_{65}B_{35}$) systems. (c) Type 3 (bcc-AB)-($A_{65}B_{35}$) systems. (d) Type 4 (fcc-Cu)-($Cu_{64}Zr_{36}$) systems. The corresponding contour maps of yield stress $\sigma_y(D, l)$ are in Figure 1c,f,i,l of the main text.

However, $\dot{\varepsilon}$ is much higher than that in real experiments [19] because low strain rates in real experiments are computationally too expensive. Excessive deformation rates may affect material strength and the deformation mechanism [20]. Nevertheless, simulations at strain rates have been well accepted as an effective method for the study of conventional HP and IHP behaviors (e.g., refs. [16, 21, 22, 23, 24]) because they can reproduce the experimental HP–IHP boundary (D^*). Different strain rates shift the $\varepsilon_y(D)$ curves but do not substantially affect the peak position, i.e. the HP–IHP boundary [25]. Our results confirm that the peak position of $\varepsilon_y(D)$ is robust under different strain rates (Figure S6a,b). Similarly, the peak position of $\varepsilon_y(l)$ is robust under different strain rates (Figure S6c,d,e).

Previous simulation studies have demonstrated that yield stress is influenced by relaxation time, temperature, and annealing time [26]. We compare the yield stresses of samples with and without annealing in Figure S6f,g. The bulk amorphous solid has been well annealed before the regions of grains are removed (Figure S1d as an example). After the amorphous GBs are combined with the crystalline regions, the whole type-1 fcc-A sample with D = 45.3 is further heated from T = 0.1 to T = 1.0 ($T_g \approx 0.8 < T < T_m \approx 1.05$ for the fcc-A crystal) over 250 τ and equilibrated at T = 1.0 for 500 τ. Afterward, the sample is cooled to T = 0.9 in 500 τ, then annealed with different cooling rates from T = 0.9 to T = 0.7 as shown in Figure S6f, and finally rapidly cooled to T = 0.1 over 500 τ and relaxed at T = 0.1 for an additional 500 τ. These samples with different annealing rates and the sample without annealing used in the main text are compared in Figure S6f. A longer annealing time shifts $\sigma_y(l)$ to higher strength but does not affect the peak position l^* (Figure S6f). In addition, Figure S6g compares the type-3 bcc-AB samples with and without annealing. The annealed sample is heated from T = 0.1 to T = 1.0 over 250 τ and relaxed at T = 1.0 for 1500 τ, then rapidly cooled to T = 0.1 in 250 τ and relaxed for another 250 τ. Figure S6g shows that the annealing has no substantial

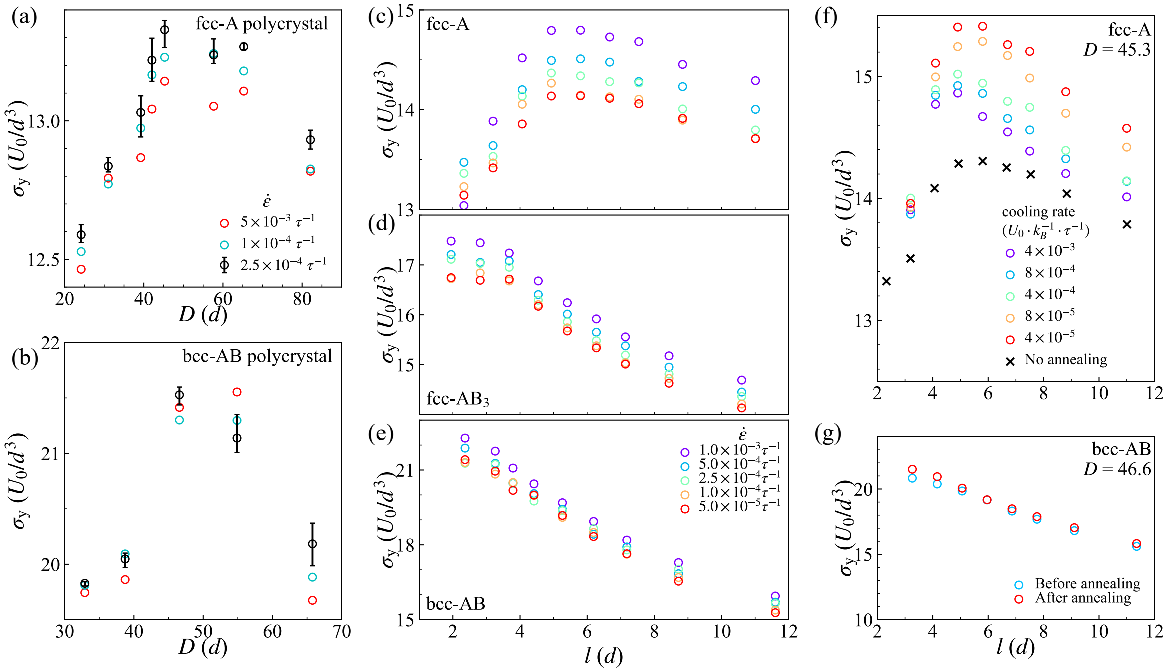

Figure S6: (a, b) Yield stress ε_y under different strain rates $\dot{\varepsilon}$ and mean grain diameters D. (a) fcc-A and (b) bcc-AB polycrystals ($l \approx 2$). (a, b) share the same legend. (c-e) Yield stress under different strain rates for (c) Type 1 fcc-A systems with $D = 45.3$, (d) Type 2 fcc-AB$_3$ systems with $D = 46.9$ and (e) Type 3 bcc-AB systems with $D = 46.6$. (c, d, e) share the same legend. (f) Yield stress of Type-1 fcc-A systems: comparison of annealed (at varying cooling rates) and non-annealed samples. (g) Yield stress of bcc-AB polycrystals before and after annealing for 3000τ under T^* .

effect on bcc samples because dislocations rarely move in bcc crystals[27].

2.4 Temperature effect on $\sigma_y(D, l)$

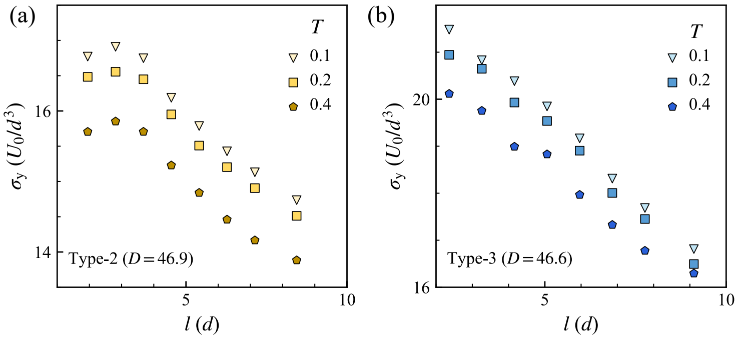

Figure S7: Temperature effect on $\sigma_y(l)$ for (a) Type 2 systems with $D = 46.9$ and (b) Type 3 systems with $D = 46.6$.

3 Mechanisms of the generalized HP and IHP behaviors

3.1 Ratio of deformations in GBs and in crystalline grains

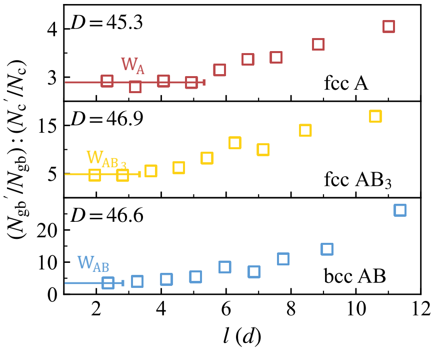

Figure S8: Ratio of the deformation intensities of the amorphous and crystalline regions. The top to bottom panels are for types 1-3 systems. N' is the number of atoms with $\eta_{\mathrm{Mises}} > 0.12$ amongst the N atoms in GBs (subscript gb) or crystals (subscript c). The horizontal lines denote the plateau widths W_{A}, $W_{\mathrm{AB_3}}$ and W_{AB}.

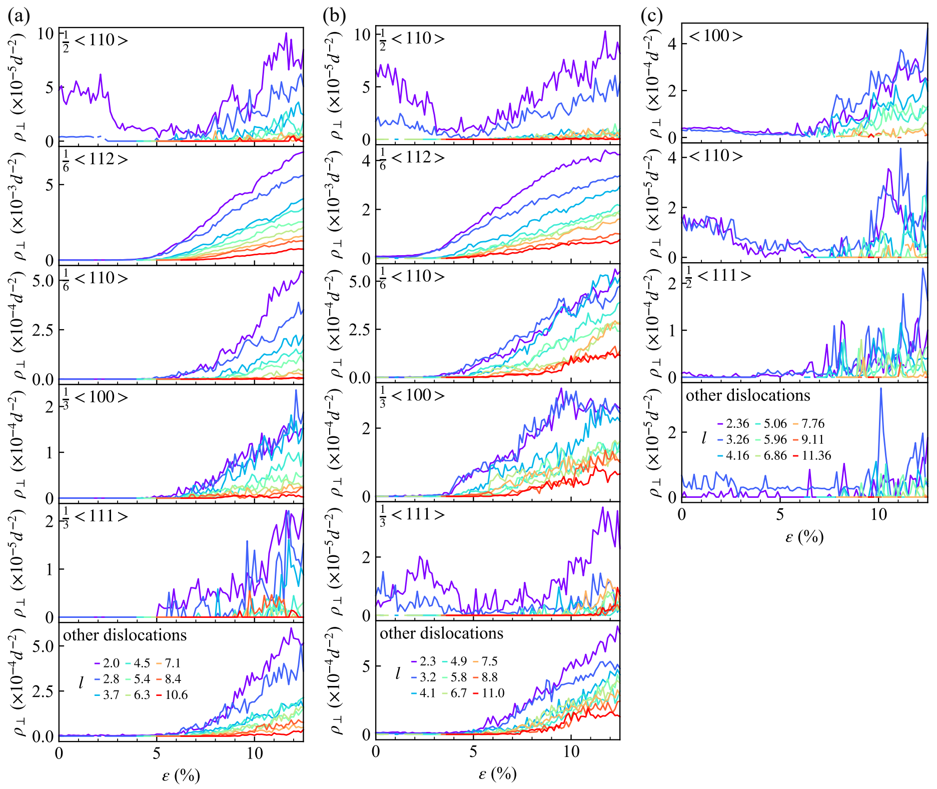

Figure S9: Densities of various types of dislocations under uniaxial strain ε. (a) Type 1 fcc systems with D = 45.3. (b) Type 2 fcc systems with D = 46.9. (c) Type 3 bcc systems with D = 46.6. Partial dislocations (e.g. $\frac{1}{6}\langle 112\rangle$) rarely exist in bcc lattices, so they are not shown in (c). The legends are in the bottom panels.

To compare the two types of deformations in the generalized HP/IHP behaviors of $\sigma_{\mathrm{y}}(D, l)$, we measure the number ratio of atoms that carry dislocation motions N_{c}' to that in all crystalline grains N_{c} and the number ratio of atom that carry GB deformations N_{gb}' to that in all amorphous GBs

N_{gb}. The ratio between the two ratios at $\sigma_{\text{y}}^{\text{max}}(l)$ (horizontal dashed lines in Figure 1c,f,i of the main text) is shown in Figure S8. A higher ratio indicates a greater atom participation ratio in plastic deformation within GB compared to the crystalline regions. While the physical interpretation of this numerical value is not immediately intuitive, its variation with respect to l reflects the transition from dislocation-motion-dominated to GB-deformation-dominated plastic deformation mechanisms. For Type 1 systems, the ratio of plastic deformation from GBs to that from crystalline regions is constant at $l < W_{\text{A}} = 6$ and increases at $l > 6$ (Figure S8). The two regimes coincide with the dislocation-motion-dominated HP-like regime and shear-transformation-zone or shear-band-dominated IHP-like regimes [3] of $\sigma_{\text{y}}(l)$ at a fixed D in Figure 1c of the main text. Shear transformation zones are localized atomic or molecular deformation patches induced by shear, and the shear bands are large zones of intense shear strain. The activation of shear transformation zones requires sufficient dislocation accumulation at GBs [28]. As l increases, dislocation density and their accumulation at GBs decrease, resulting in a decrease in $N_{\text{c}}'(l)$ and a slower growth of $N_{\text{GB}}'(l)$ compared to $N_{\text{GB}}(l)$. Consequently, both $N_{\text{c}}'/N_{\text{c}}$ and $N_{\text{GB}}'/N_{\text{GB}}$ decrease, leaving the ratio $(N_{\text{GB}}'/N_{\text{GB}}) : (N_{\text{c}}'/N_{\text{c}})$ unchanged. In contrast, when $l > l^*$, GBs can readily deform via shear banding even at low strains insufficient to activate significant dislocation motions. This leads to an increase in $N_{\text{GB}}'(l)/N_{\text{GB}}(l)$ which dominates $(N_{\text{GB}}'/N_{\text{GB}}) : (N_{\text{c}}'/N_{\text{c}})$. The dislocation-dominated-deformation regime is at $l < W_{\text{B}} = 3$ for Type 2 systems, which is narrower than the $l < 6$ regime for type 1 systems because Type 2 systems have fewer dislocations (Figure 2e of the main text). Type 3 bcc systems are nearly dislocation free (Figure 2f of the main text), so GB deformation dominates and $(N_{\text{gb}}'/N_{\text{gb}}) : (N_{\text{c}}'/N_{\text{c}})$ in Figure S8 monotonically increases with l. The plateau regimes at $l < W = 6, 3, 2$ atom in Figure S8 correspond well to $l^* \approx 6, 3, 2$ at $\sigma_{\text{y}}^{\text{max}}$ (☆ in Figure 1c,f,i of the main text). This result confirms the dislocation-motion-dominated and GB-dominated deformation mechanisms in the increasing and decreasing regimes of $\sigma_{\text{y}}^{\text{max}}(l)$, respectively. In summary, the plateau width $W_{\text{fcc-A}} > W_{\text{fcc-AB}_3} > W_{\text{bcc-AB}}$ of Figure S8 is in accordance with $\rho_{\perp}^{\text{fcc-A}} > \rho_{\perp}^{\text{fcc-AB}_3} > \rho_{\perp}^{\text{bcc-AB}}$ in Figure 2d–f,h of the main text and explains $l^*_{\text{fcc-A}} > l^*_{\text{fcc-AB}_3} > l^*_{\text{bcc-AB}}$ in Figure 1c,f,i of the main text.

3.2 Dislocation density during deformation

Different types of dislocations and their densities are measured using the dislocation analysis method [29]. The densities of different types of dislocations during the deformation process are shown in Figure S9. In fcc Type 1 (Figure S9a) and Type 2 (Figure S9b) systems, the predominant dislocations are highly mobile $\frac{1}{6}\langle 112\rangle$ (Shockley) partial dislocations and $\frac{1}{6}\langle 110\rangle$ (stair-rod) dislocations. In bcc crystals, edge and partial dislocations have high activation energies and thus rarely exist [27, 30]. Screw dislocation is favorable for bcc crystals [27]. However, these screw dislocations are non-planar and thus exhibit high gliding resistance and low mobility [27]. This is confirmed by the absence of edge dislocations in Type 3 bcc systems (Figure S9c) during the flow stage after yielding ($\varepsilon > 6\%$). Therefore, the plastic deformations of bcc composites are mainly in GBs instead of in crystalline grains via dislocation motions.

3.3 Required stress to create a dislocation in different crystals

The energy of a stacking fault in Figure 2i of the main text is measured as the potential energy difference between two single crystals with and without the stacking fault [31]. The stacking fault is shown in Figure S10 and produced as follows. After an energy minimization process under $10P^*$, we displace the upper part of the defect-free crystal slightly along the $[\bar{1}\bar{2}1]$ direction and set atom to be movable only in the z direction (Figure S10), followed by another energy minimization.

We compare the critical stresses of creating a dislocation in three types of defect-free single crystals under the same temperature and pressure. Given that a defect-free crystal yields right after the formation of the first dislocation, the yield stress of a defect-free crystal (i.e. maximum of each $\sigma(\varepsilon)$ in Figure 2j of the main text) is used as the stress required for the generation of dislocation [32]. We observe that a screw dislocation is always generated first in the bcc crystal, and an edge dislocation is always generated first in the fcc crystal at a low stress (Figure 2j of the main text). These results are consistent with the fact that plastic deformations are dominated by screw dislocations in bcc crystals and by edge dislocations in fcc crystals [27] because creating a screw dislocation requires much higher stress than that required for edge dislocation in fcc crystals [27].

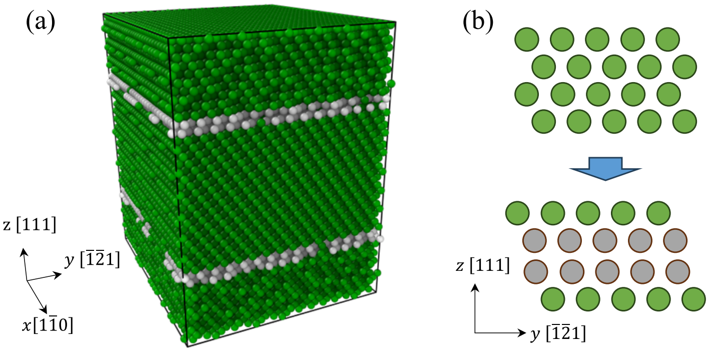

Figure S10: (a) Two stacking faults (grey) along the (111) slip plane in an fcc crystal under periodic boundary conditions. (b) Cross-section on the y-z plane showing that a stacking fault can be created by slightly shifting half of a perfect crystal.

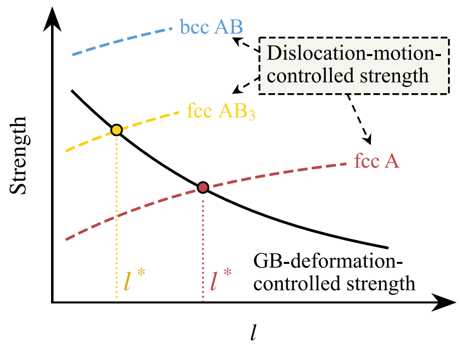

Figure S11: Illustration of solid strength as a function of l under different deformation mechanisms. The dashed curves represent dislocation-motion-controlled strength. The activation stress or friction stress σ_0 of dislocation motion is much higher in the bcc lattice than in the fcc lattice, thus the dislocation-motion-controlled strength (dashed curves) is high for the bcc lattice. GB deformation is not influenced by different types of lattices, so the GB-controlled strength curve does not shift.

The activation stress for plastic deformation (i.e. dislocation motions) in crystalline grains depends on the composition and lattice structure, but not on l. By contrast, the activation stress for plastic deformation in GBs decreases with l but is independent of the composition and lattice structure of crystalline grains. These results are illustrated in Figure 2k of the main text. In addition, the strength depends on both σ_0 and dislocation density. Thick-GB samples contain minimal dislocations, so higher applied stress is needed to achieve the same amount of deformation, leading to the dislocation-motion-controlled strength for the crystalline parts increasing with l. The combination of this result with those in Figure 2k provides a sketch of dislocation-motion-controlled strength and GB-deformation-controlled strength as a function of l (shown in Figure S11). When one type of strength (e.g., dislocation-motion-controlled) is lower than the other, i.e., this type of deformation is easier, then it will proliferate and largely pre-empt the other type (e.g. GB-controlled) of deformation.

3.4 Plastic deformations are more uniform in thicker GBs

The uniformity of strain in GBs at the particle length scale can be characterized by the relative standard deviation of η_{Mises} [33], namely, $\text{RSD}^{\text{GB}}_{\eta_{\text{Mises}}} = \sqrt{\sum_i^{N_{\text{GB}}}(\eta_i - \bar{\eta})^2/N_{\text{GB}}}$, where η_i is the shear strain of particle i and $\bar{\eta}$ is the average over atom in GBs. N_{GB} is the number of atom in GBs. The measured $\text{RSD}^{\text{GB}}_{\eta_{\text{Mises}}}$ decreases with l regardless of the composition in the crystalline region (Figure S12), indicating that deformations are more uniform at the single-particle scale in thicker GBs.

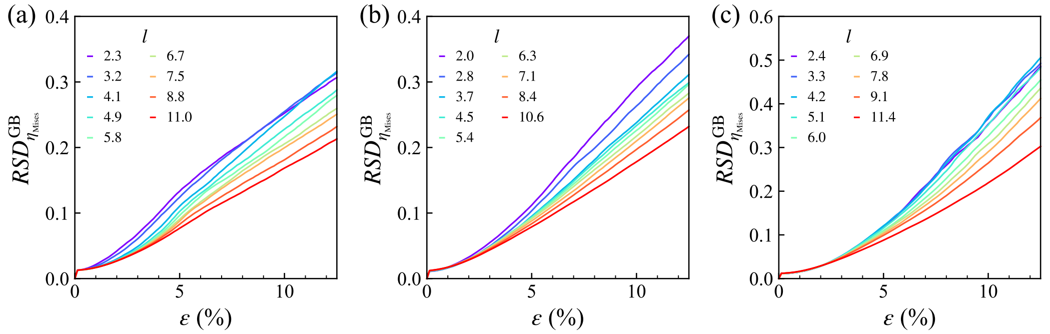

Figure S12: Relative standard deviation (RSD) of von Mises shear strain η_{Mises} of atom in GBs during deformation. (a) Type 1 systems with D = 42.05. (b) Type 2 systems with D = 46.49. (c) Type 3 systems with D = 46.57.

4 Fracture process

Fracture behavior, whether brittle or ductile, can be identified by fracture morphology. Fractures exhibit various morphologies depending on material properties [34], such as ductile fracture featured with necking or shear deformation and brittle facture featured with cracking or brittle shear [35]. To our knowledge, the fracture process and fracture morphology have not been explored using crystalline–amorphous composites.

Figure S13 shows different types of ductile fractures in three fcc composites with the same D and different l under the tensile deformation along the z direction. Their stress–strain curves are shown in Figure 4a of the main text. For thin-GB samples (e.g. l = 2.8 in Figure S13a,b), necking occurs, that is, a prominent decrease in the cross-sectional area occurs. Necking is caused by a local stress concentration with a large amount of strain hardening [36]. We observe that dislocations move into GBs, thus inducing stresses at GBs (Figure S13a,b). When ε reaches 10%, a crack initiates from a free surface on the yz plane and rapidly propagates along the nearby GBs, resulting in sudden failure at $\varepsilon \approx$ 20%. For the sample with l = 6 in Figure S13c,d, similar dislocation motions induce necking, followed by shear deformation that starts from the necking region, penetrates through GBs and splits the sample (Movie S5). By contrast, plastic deformation in the thick-GB samples (e.g. l = 18 in Figure S13e,f) occurs solely within thick GBs without obvious necking because few dislocations or stacking faults are produced in crystalline grains during tensile deformation. Thus, GB deformation occurs easily, which pre-empts necking. The shear plane also initiates from a free surface and penetrates through thick GBs, resulting in fracture.

Figure S14 shows the ductile and brittle fractures of three bcc composites with different (D, l). Their stress–strain curves are shown in Figure 4k,l of the main text. The deformation process of the bcc polycrystal ($l \approx 3$) changes from brittle to ductile behavior as D decreases. The small-D samples undergo ductile shear deformation before ε reaches 10%, and the crack propagates along the GBs and splits the sample, leading to fracture (Figure S14a,b). By contrast, the large-D samples (Figure S14c,d) exhibit no substantial plastic deformation within the grains. When ε increases to about 4%, a crack initiates from a triple junction inside the bulk instead of from a free surface. It rapidly propagates along the GBs, producing a void followed by a rough fracture surface (Figure S14c,d). The fracture of the thick-GB bcc samples (Figure S14e,f) is similar to that observed in the thick-GB fcc samples (Figure S13e,f): plastic deformations occur solely within the thick GBs. Bcc crystals exhibit higher friction stresses for dislocation motions than fcc crystals [27]. These elevated friction stresses in bcc composites enhance their resistance to amorphization [37], which impedes shear plane extension in bcc systems and promotes the formation of additional shear planes (yellow ellipse in Figure S14f), resulting in multi-plane shear (i.e., slipping off) [38]. According to Schmid's law, the secondary shear plane in the amorphous phase is typically perpendicular to the initial shear plane [39]. Such a perpendicular secondary shear plane is clearly observed (Figure S14f) in our systems, which produces a necking-like deformation (Figure S14e,f).

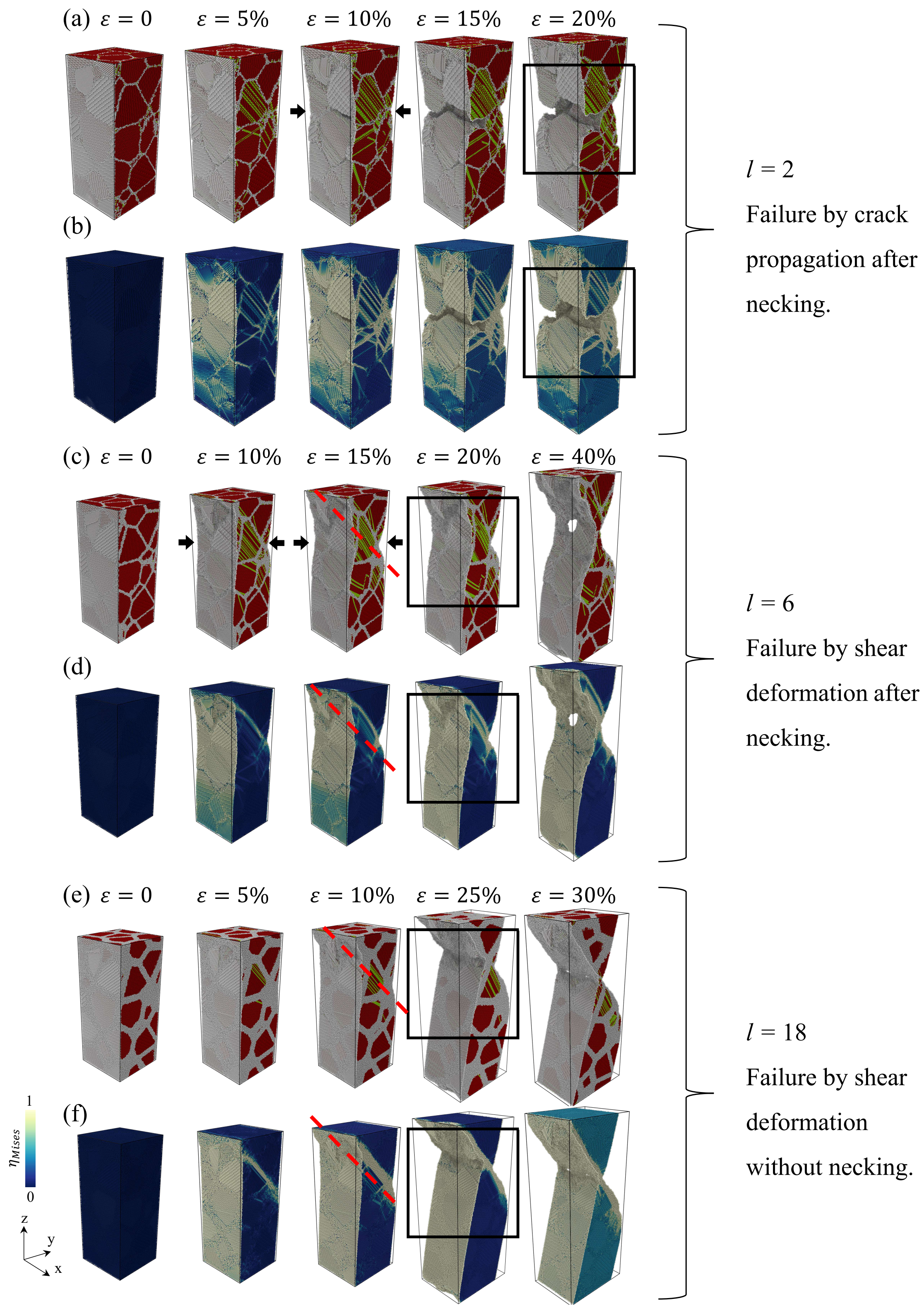

Figure S13: Fracture processes of three fcc samples with D = 45.1 on the dashed line in Figure 1c of the main text. (a and b) l = 2.8, (c and d) l = 6.0, (e and f) l = 18.0. Each particle is colored by its local structure (red: fcc; green: hcp; grey: amorphous) in (a, c, and e) and by η_{Mises} in (b, d, and f). The pairs of black arrows in (a and c) denote necking. The red dashed lines denote shear deformation. The black boxes in (a to f) mark the regions shown in Figure 4c–h of the main text, respectively.

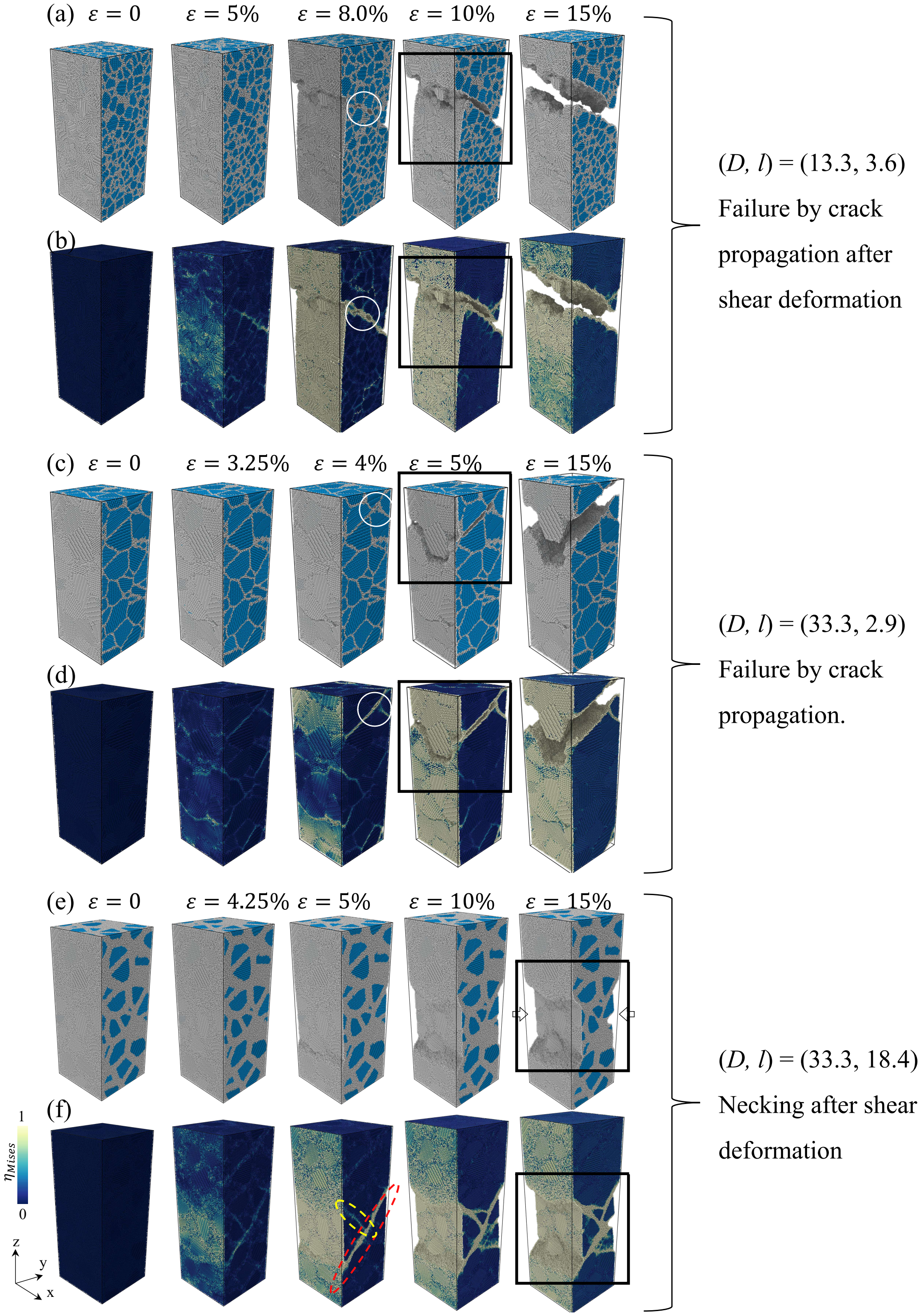

Figure S14: Fracture processes of three bcc samples. (a and b) $(D, l) = (13.3, 3.6)$, (c and d) $(D, l) = (33.3, 2.9)$, (e and f) $(D, l) = (33.3, 18.4)$. Each particle is colored by its local structure (blue: bcc; grey: amorphous) in (a, c, and e) and by η_{Mises} in (b, d, and f). The red and yellow ellipses in (f) mark two perpendicular shear planes with $\pm 45^{\circ}$ relative to the stretch in the z direction. The white circles in (a to d) mark the voids, which lead to a rough fracture surface. The black boxes in (a to f) mark the regions shown in Figure 4m–r of the main text, respectively.

5 Elastic Moduli

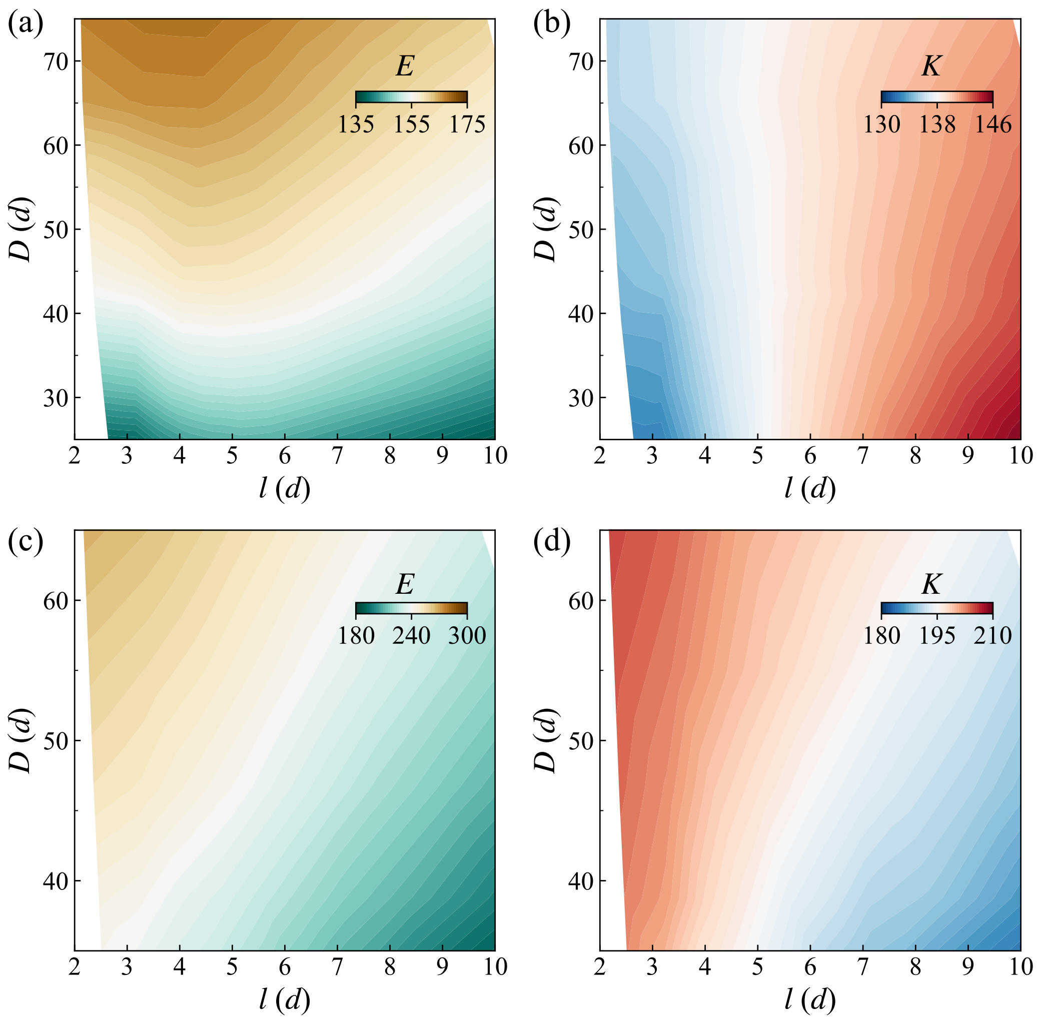

Figure S15: Contour maps of elastic moduli of Type-1 systems (top panels) and Type-3 systems (bottom panels). (a and c) Young's moduli $E(D, l)$. (b and d) Bulk moduli $K(D, l)$.

The elastic moduli in the (D, l) space are shown in Figure S15. Figure S15a,c demonstrates that increasing D enhances E in both fcc Type-1 and bcc Type-3 systems, consistent with the trend in polycrystals [40]. E peaks at $l \approx 5$ in Type-1 systems, but decreases linearly with l in Type-3 systems. This contrast can be explained as follows. As l increases, the GB fraction increases, while the fractions of crystalline regions and GB-crystal interfacial layers shrink. For Type-3 systems, the amorphous GB has a lower E than the crystal and interfacial layers (Figure 5c in the main text), as the glass phase under high strain rates behaves similarly to a liquid. Consequently, E of the whole sample decreases monotonically with l (Figure S15c). By contrast, for Type-1 systems, the amorphous GB exhibits intermediate E, lower than the crystal but higher than the interfacial layer (Figure 5a in the main text), resulting in the non-monotonic $E(l)$ in Figure S15a. The interfacial layers consist of pure A particles in Type-1 systems and $A_{50}B_{50}$ in Type-3 systems. Thick GBs are $A_{65}B_{35}$ in both types of systems. Since B particles have stronger attractions than A particles, higher B-fraction regions (e.g., GBs in Type-1 systems) exhibit larger E and K. Figure S15b shows that $K(D, l)$ increases with D at large l, but decreases at small l for Type-1 systems. This is because $K_{\text{interfacial}} < K_{\text{crystal}} < K_{\text{GB}}$, as shown in Figure 5b of the main text. There are two interfacial layers with a 1.5-particle thickness and an $l - 2 \times 1.5$ thick amorphous GB layer. Therefore, increasing D raises the interfacial fraction more than the GBs' fraction at $l < 6$, and this trend reverses at $l > 6$. For Type-3 systems, both

$K_{\text{interfacial}}$ and K_{GB} are smaller than K_{crystal} (Figure 5d of the main text). Both increasing D and decreasing l reduce the amorphous fraction, and consequently increase K (Figure S15d). These results provide guidance for optimizing elastic moduli in crystalline-amorphous composites by tailoring (D, l) parameters.

6 Movies

Movie S1: Compression processes of two Type 1 systems (fcc A)-($A_{65}B_{35}$). Top row: solid with the maximum strength, i.e. the ☆ in Figure 1c with $(D, l) = (45.3, 5.8)$. Panels from left to right: stress–strain curve, the system with each particle colored by its local order (amorphous, fcc, or hcp lattice), the system with each particle colored by its von Mises shear strain and dislocations. Bottom row: results for the solid with $(D, l) = (45.3, 11.0)$.

Movie S2: Compression process of two Type 2 systems (fcc AB_3)-($A_{65}B_{35}$). Top row: solid with the maximum strength, i.e. the ☆ in Figure 1f of the main text with $(D, l) = (46.9, 2.8)$. Panels from left to right: stress–strain curve, the system with each particle colored by its local order (amorphous, fcc, or hcp lattice), the system with each particle colored by its von Mises shear strain and dislocations. Bottom row: results for the solid with $(D, l) = (46.9, 10.6)$.

Movie S3: Compression process of two Type 3 systems (bcc AB)-($A_{65}B_{35}$). Top row: solid with the maximum strength, i.e. the ☆ in Figure 1i of the main text with $(D, l) = (46.6, 2.4)$. Panels from left to right: stress–strain curve, the system with each particle colored by its local order (amorphous, bcc lattice), the system with each particle colored by its von Mises shear strain and dislocations. Bottom row: results for the solid with $(D, l) = (46.6, 11.4)$.

Movie S4: Tensile deformation process of the Type 1 system (fcc A)-($A_{65}B_{35}$) with $(D, l) = (45.1, 2.8)$. A snapshot is shown in Figure 4c,f of the main text. Panels from left to right: stress–strain curve, the system with each particle colored by its local order (amorphous, fcc, or hcp lattice) and the system with each particle colored by its von Mises shear strain.

Movie S5: Tensile deformation process of the Type 1 system (fcc A)-($A_{65}B_{35}$) with $(D, l) = (45.1, 6.0)$. A snapshot is shown in Figure 4d,g of the main text. Panels from left to right: stress–strain curve, the system with each particle colored by its local order (amorphous, fcc, or hcp lattice) and the system with each particle colored by its von Mises shear strain.

Movie S6: Tensile deformation process of the Type 1 system (fcc A)-($A_{65}B_{35}$) with $(D, l) = (45.1, 18)$. A snapshot is shown in Figure 4e,h of the main text. Panels from left to right: stress–strain curve, the system with each particle colored by its local order (amorphous, fcc, or hcp lattice), and the system with each particle colored by its von Mises shear strain.

Movie S7: Tensile deformation process of the Type 3 system (bcc AB)-($A_{65}B_{35}$) with $(D, l) = (13.2, 3.6)$. A snapshot is shown in Figure 4m,p of the main text. Panels from left to right: stress–strain curve, the system with each particle colored by its local order (amorphous or bcc lattice), and the system with each particle colored by its von Mises shear strain.

Movie S8: Tensile deformation process of the Type 3 system (bcc AB)-($A_{65}B_{35}$) with $(D, l) = (33.3, 2.9)$. A snapshot is shown in Figure 4n,q of the main text. Panels from left to right: stress–strain curve, the system with each particle colored by its local order (amorphous or bcc lattice), and the system with each particle colored by its von Mises shear strain.

Movie S9: Tensile deformation process of the Type 3 system (bcc AB)-($A_{65}B_{35}$) with $(D, l) = (33.3, 18.4)$. A snapshot is shown in Figure 4o,r of the main text. Panels from left to right: stress–strain curve, the system with each particle colored by its local order (amorphous or bcc lattice), and the system with each particle colored by its von Mises shear strain.

References

[1] Brink, T. & Albe, K. From metallic glasses to nanocrystals: molecular dynamics simulations on the crossover from glass-like to grain-boundary-mediated deformation behaviour. Acta Mater. **156**, 205–214 (2018).

[2] Xiao, J. & Deng, C. Mitigating the Hall–Petch breakdown in nanotwinned Cu by amorphous intergranular films. Scripta Mater. **194**, 113682 (2021).

[3] Qian, L. et al. Amorphous thickness-dependent strengthening–softening transition in crystalline–amorphous nanocomposites. Nano Lett. **23**, 11288-11296 (2023).

[4] Hirel, P. Atomsk: A tool for manipulating and converting atomic data files. Comput. Phys. Commun. **197**, 212–219 (2015).

[5] Edelsbrunner, H. & Mücke, E. P. Three-dimensional alpha shapes. ACM Trans. Graph. **13**, 43–72 (1994).

[6] Stukowski, A. Visualization and analysis of atomistic simulation data with ovito–the open visualization tool. Model. Simul. Mater. Sci. **18**, 015012 (2009).

[7] Brüning, R., St-Onge, D. A., Patterson, S. & Kob, W. Glass transitions in one-, two-, three-, and four-dimensional binary Lennard-Jones systems. J. Phys. Condens. Matter **21**, 035117 (2008).

[8] Plimpton, S. Fast parallel algorithms for short-range molecular dynamics. J. Comput. Phys. **117**, 1–19 (1995).

[9] Kob, W. & Andersen, H. C. Testing mode-coupling theory for a supercooled binary Lennard-Jones mixture I: The van Hove correlation function. Phys. Rev. E **51**, 4626 (1995).

[10] Mendelev, M., Sun, Y., Zhang, F., Wang, C.-Z. & Ho, K.-M. Development of a semi-empirical potential suitable for molecular dynamics simulation of vitrification in Cu-Zr alloys. J. Chem. Phys. **151** (2019).

[11] Honeycutt, J. D. & Andersen, H. C. Molecular dynamics study of melting and freezing of small Lennard-Jones clusters. J. Phys. Chem. **91**, 4950–4963 (1987).

[12] Mises, R. v. Mechanik der plastischen formänderung von kristallen. ZAMM Z. Angew. Math. Mech. **8**, 161–185 (1928).

[13] Lubliner, J. Plasticity Theory (Courier Corporation, 2008).

[14] Clavier, G. et al. Computation of elastic constants of solids using molecular simulation: comparison of constant volume and constant pressure ensemble methods. Mol. Simul. **43**, 1413–1422 (2017).

[15] Li, Z. & Bradt, R. C. The single-crystal elastic constants of cubic (3c) SiC to 1000 C. J. Mater. Sci. **22**, 2557–2559 (1987).

[16] Schiøtz, J. & Jacobsen, K. W. A maximum in the strength of nanocrystalline copper. Science **301**, 1357–1359 (2003).

[17] Dowding, I. & Schuh, C. A. Metals strengthen with increasing temperature at extreme strain rates. Nature **630**, 91–95 (2024).

[18] Lee, J.-H. et al. High strain rate deformation of layered nanocomposites. Nat. Commun. **3**, 1164 (2012).

[19] Gu, J., Duan, F., Liu, S., Cha, W. & Lu, J. Phase engineering of nanostructural metallic materials: Classification, structures, and applications. Chem. Rev. **124**, 1247–1287 (2024).

[20] Fan, Y., Osetsky, Y. N., Yip, S. & Yildiz, B. Onset mechanism of strain-rate-induced flow stress upturn. Phys. Rev. Lett. **109**, 135503 (2012).

[21] Wolf, D., Yamakov, V., Phillpot, S., Mukherjee, A. & Gleiter, H. Deformation of nanocrystalline materials by molecular-dynamics simulation: relationship to experiments? Acta Mater. **53**, 1–40 (2005).

[22] Bringa, E. M. et al. Ultrahigh strength in nanocrystalline materials under shock loading. Science **309**, 1838–1841 (2005).

[23] Zepeda-Ruiz, L. A., Stukowski, A., Oppelstrup, T. & Bulatov, V. V. Probing the limits of metal plasticity with molecular dynamics simulations. Nature **550**, 492–495 (2017).

[24] Cao, P. Maximum strength and dislocation patterning in multi–principal element alloys. Sci. Adv. **8**, eabq7433 (2022).

[25] Carlton, C. & Ferreira, P. What is behind the inverse Hall–Petch effect in nanocrystalline materials? Acta Mater. **55**, 3749–3756 (2007).

[26] Valiev, R. Z., Islamgaliev, R. K. & Alexandrov, I. V. Bulk nanostructured materials from severe plastic deformation. Prog. Mater. Sci. **45**, 103–189 (2000).

[27] Hull, D. & Bacon, D. J. Introduction to Dislocations, vol. 37 (Elsevier, 2011).

[28] Wang, Y., Li, J., Hamza, A. V. & Barbee Jr, T. W. Ductile crystalline–amorphous nanolaminates. Proc. Natl. Acad. Sci. **104**, 11155–11160 (2007).

[29] Stukowski, A., Bulatov, V. V. & Arsenlis, A. Automated identification and indexing of dislocations in crystal interfaces. Model. Simul. Mater. Sci. **20**, 085007 (2012).

[30] Greer, J. R., Weinberger, C. R. & Cai, W. Comparing the strength of fcc and bcc sub-micrometer pillars: compression experiments and dislocation dynamics simulations. Mater. Sci. Eng. A **493**, 21–25 (2008).

[31] Van Swygenhoven, H., Derlet, P. M. & Frøseth, A. Stacking fault energies and slip in nanocrystalline metals. Nat. Mater. **3**, 399–403 (2004).

[32] Salehinia, I. & Bahr, D. Crystal orientation effect on dislocation nucleation and multiplication in fcc single crystal under uniaxial loading. Int. J. Plasticity **52**, 133–146 (2014).

[33] Cheng, Y., Cao, A. & Ma, E. Correlation between the elastic modulus and the intrinsic plastic behavior of metallic glasses: the roles of atomic configuration and alloy composition. Acta Mater. **57**, 3253–3267 (2009).

[34] Passchier, C. W. & Trouw, R. A. Microtectonics (Springer Science & Business Media, 2005).

[35] Gdoutos, E. E. Fracture mechanics: An Introduction, vol. 263 (Springer Nature, 2020).

[36] Knott, J. F. Fundamentals of Fracture Mechanics (Gruppo Italiano Frattura, 1973).

[37] Madec, R. & Kubin, L. P. Dislocation strengthening in fcc metals and in bcc metals at high temperatures. Acta Mater. **126**, 166–173 (2017).

[38] Noell, P. J., Carroll, J. D. & Boyce, B. L. The mechanisms of ductile rupture. Acta Mater. **161**, 83–98 (2018).

[39] Budrikis, Z., Castellanos, D. F., Sandfeld, S., Zaiser, M. & Zapperi, S. Universal features of amorphous plasticity. Nat. Commun. **8**, 15928 (2017).

[40] Meyers, M. A., & Chawla, K. K. Mechanical Behavior of Materials (Cambridge University Press, 2008).
